# LncRNA-p21 alters the antiandrogen enzalutamide-induced prostate cancer neuroendocrine differentiation via modulating the EZH2/STAT3 signaling

**DOI:** 10.1038/s41467-019-09784-9

**Published:** 2019-06-12

**Authors:** Jie Luo, Keliang Wang, Shuyuan Yeh, Yin Sun, Liang Liang, Yao Xiao, Wanhai Xu, Yuanjie Niu, Liang Cheng, Sankar N. Maity, Runze Jiang, Chawnshang Chang

**Affiliations:** 1 George Whipple Lab for Cancer Research, Departments of Pathology, Urology, Radiation Oncology, Biology and The Wilmot Cancer Institute, University of Rochester, Rochester, NY 14642 USA; 2 Department of Urology, The 4th Affiliated Hospital of Harbin Medical University, Harbin, 150001 China; 3 Department of Urology, Shanxi Province People’s Hospital, Xi’an, 710068 Shanxi China; 4 Tianjin Institute of Urology, Tianjin Medical University, Tianjin, 300211 China; 5 Department of Pathology & Laboratory Medicine, Indiana University, Indianapolis, 46202 IN USA; 6 Department of Genitourinary Medical Oncology, The University of Texas MD Anderson Cancer Center, Houston, 77030 TX USA; 7 Jiangmen Maternity and Child Health Care Hospital, Jiangmen, 529000 Guangdong China; 8 Sex Hormone Research Center, China Medical University and Hospital, Taichung, 404 Taiwan

**Keywords:** Urological cancer, Prostate cancer

## Abstract

While the antiandrogen enzalutamide (Enz) extends the castration resistant prostate cancer (CRPC) patients’ survival an extra 4.8 months, it might also result in some adverse effects via inducing the neuroendocrine differentiation (NED). Here we found that lncRNA-p21 is highly expressed in the NEPC patients derived xenograft tissues (NEPC-PDX). Results from cell lines and human clinical sample surveys also revealed that lncRNA-p21 expression is up-regulated in NEPC and Enz treatment could increase the lncRNA-p21 to induce the NED. Mechanism dissection revealed that Enz could promote the lncRNA-p21 transcription via altering the androgen receptor (AR) binding to different androgen-response-elements, which switch the EZH2 function from histone-methyltransferase to non-histone methyltransferase, consequently methylating the STAT3 to promote the NED. Preclinical studies using the PDX mouse model proved that EZH2 inhibitor could block the Enz-induced NED. Together, these results suggest targeting the Enz/AR/lncRNA-p21/EZH2/STAT3 signaling may help urologists to develop a treatment for better suppression of the human CRPC progression.

## Abstract (web summary)

The induction of neuroendocrine differentiation occurs in enzalutamide treated castration resistant prostate cancer. Here, the authors show that lncRNA-21 mediates enzalutamide induced neuroendocrine differentiation through EZH2/STAT axis and EZH2 inhibition suppresses this differentiation.

## Introduction

Prostate cancer (PCa) is the most prevalent male cancer in the U.S^1^, and the current standard androgen deprivation therapy (ADT) with the FDA-approved antiandrogen Enzalutamide (Enz, also known as MDV3100) may effectively suppress the castration resistant PCa (CRPC) and extend CRPC patients survival an extra 4.8 months. However, ADT with Enz treatment (ADT-Enz) may also induce some unwanted adverse effects including development of Enz-resistance and increasing the PCa cell invasion as demonstrated in several preclinical models^2,3^, as well as promoting the neuroendocrine (NE) differentiation (NED)^4,5^.

The increase of neuroendocrine PCa cells (NEPC) represents a severe condition during ADT because these NEPC cells express little androgen receptor (AR) and will not respond to current ADTs^6^. Importantly, NEPC can also function via secreting cytokines or growth factors to stimulate the PCa growth^7^, and increase the surrounding PCa cells resistance to chemotherapy^8^. Moreover, knocking down AR in the PCa cells could also increase the expression of NE markers^5,8^. The detailed mechanisms, however, remain unclear.

The AR is the master regulator to modulate PCa progression. While most ADTs can either reduce the androgen production or prevent androgens from binding to AR, ADT may continue to increase the expression of AR, even at the castration-resistant stage^9,10^, and AR may be transactivated via several non-androgens factors, including growth factors, cytokines and kinases^11^. Importantly, AR may also play dual roles to either function as a proliferator to enhance the PCa cell growth or function as suppressor of PCa cell invasion as demonstrated in several preclinical in vitro cell lines and in vivo mouse models^3,12–14^.

Recently, accumulating evidence suggested that long non-coding RNAs (lncRNAs) might play key roles to regulate cancer progression^15^. The lncRNA-p21 was identified as the p53 co-repressor^16^, and might activate p21 to promote the PRC2 target genes expression to impact the cellular epigenetic regulation^17^. Other studies also suggested that lncRNA-p21 could function as a biomarker to monitor the PCa progression^18^.

The histone methyltransferase Enhancer of Zeste Homolog 2 (EZH2), can function as the enzymatic core component of Polycomb Repressive Complex 2 (PRC2) to catalyze the Histone 3 lysine 27 tri-methylation for inhibition of the downstream genes transcription at the epigenetic level^19^. EZH2 can also function as a non-histone methyltransferase independent of the PRC2 complex^20,21^ to methylate non-histone proteins, such as STAT3 and AR, thus promoting tumorigenicity^22,23^. However, the detailed mechanisms to switch the EZH2 histone to non-histone methyltransferase function are unclear. Interestingly, in CRPC cells, the oncogenic activity of EZH2 does not rely on the PRC2 complex, suggesting that this EZH2 PRC2 independent function is also critical in CRPC cells. Importantly, much higher expression of EZH2 was also found in the NEPC cells^24,25^. And recent studies suggested that a high expression of EZH2 can alter the epigenetic programming in NEPC cells and promote the NEPC cells growth^26,27^. Such regulation still relies on EZH2 PRC2 dependent function. The detailed mechanisms to link the ADT-Enz to altering the EZH2 expression to impact the NED, however, remain unclear.

Here we found that Enz might function via altering the AR/lncRNA-p21/EZH2/STAT3 axis and targeting this signal with an EZH2 inhibitor can reduce the Enz treatment-unwanted adverse effect of promoting the NED.

## Results

### lncRNA-p21 expression is increased in NEPC cells

Early studies indicated that Enz might induce some adverse-effects, including enhancing the NED, which may then promote the Enz-resistance^5^. The detailed mechanisms, especially its potential linkage to the lncRNAs, key players in cancer progression^28^, however, remain unclear.

**Fig. 1:**
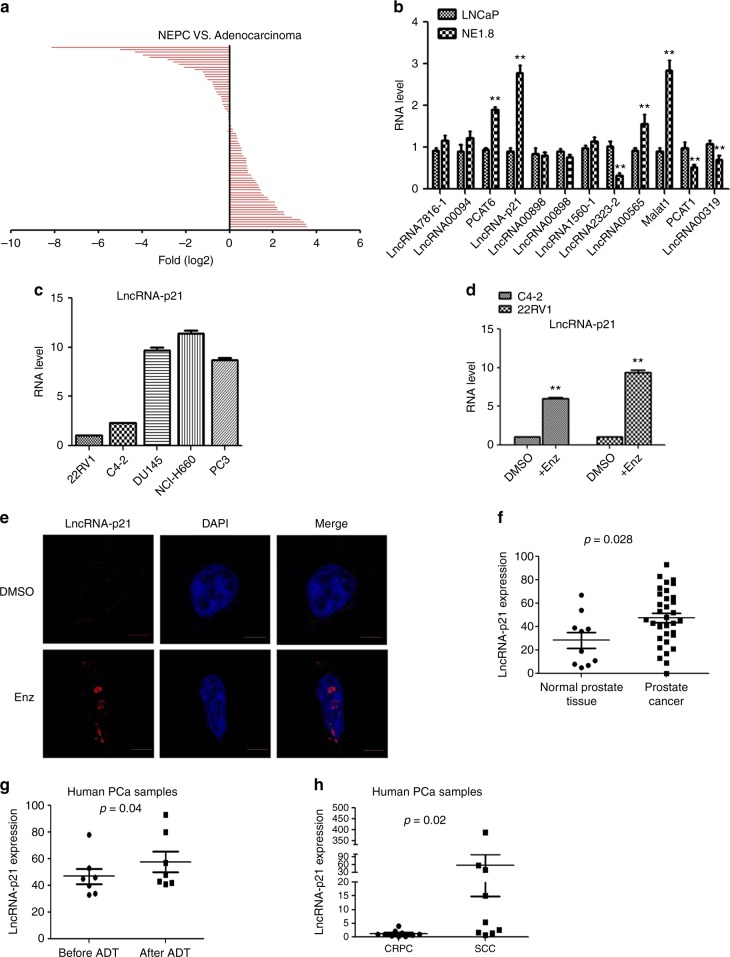
The lncRNA-p21 level is increased in NEPC cells. **a** The qPCR analysis to detect 75 lncRNAs expressed in NEPC-PDX and adenocarcinoma-PDX samples. **b** Detecting lncRNAs expressions in LNCaP and NE1.8 cells by qPCR analysis. **c** The qPCR analysis of lncRNA-p21 expression in CWR22RV1 (22RV1), C4-2, DU145, NCI-H660, and PC3 cells. **d** The qPCR analysis of lncRNA-p21 expression in C4-2 and 22RV1 cells after Enz treatment. **e** Detecting the lncRNA-p21 expression in Enz-treated C4-2 cells by RNA in situ hybridization (Scale bar = 2 μm). **f** The lncRNA-p21 expression in human normal prostate tissues (*n* = 10) and human PCa tissues (*n* = 34). **g** The lncRNA-p21 expression in PCa tissues before and after ADT (*n* = 7). **h** Detection of lncRNA-p21 level in human CRPC (*n* = 10) and PCa small cell carcinoma (SCC) (*n* = 10) samples. For B and D, data are presented as mean ± SD, **p < 0.005, by *t-*test

We first screened 75 lncRNAs whose expressions are correlated with PCa progression in the PCa adenocarcinoma PDX (PDX-133-4C) and NEPC PDX (PDX-144-13C) xenograft studies^29,30^, and results revealed that many lncRNAs expression were significantly increased in the NEPC-PDX samples (Fig. 1a and Supplementary Data 1). We then detected 12 lncRNAs whose expression were most significantly increased in the NEPC-PDX samples as compared to the adenocarcinoma-PDX samples, and identified the highest expression of lncRNA-p21 in NE1.8 cells compared to LNCaP cells (Fig. 1b). Furthermore, we also found the higher expression of lncRNA-p21 in DU145 (NE-like cells)^31,32^ NCI-H660 (NEPC)^24^ cells, and PC3 cells (NE-like cells) as compared to that found in the CWR22RV1 and C4-2 cells (Fig. 1c).

Importantly, using both qPCR and In Situ hybridization, we found treating with Enz could increase the lncRNA-p21 expression in both C4-2 and CWR22RV1 cells, suggesting that lncRNA-p21 might play a key role in the Enz-induced NED (Fig. 1d, e)

We then examined the expression of lncRNA-p21 in human clinical samples from two published RNA sequencing database^33,34^ and results revealed higher lncRNA-p21 expression in PCa samples than in normal prostate tissues (Fig. 1f), as well as higher expression in the PCa patients who received the ADT (Fig. 1g and Supplementary Fig. 1B). Importantly, we assayed the human clinical samples and found that lncRNA-p21 expression was increased significantly in small cell carcinoma (SCC) samples compared to CRPC samples (Fig. 1h).

Together, results from Fig. 1a–h suggest that lncRNA-p21 expression is increased in the NEPC cells.

### lncRNA-p21 promotes the NED after Enz treatment

**Fig. 2:**
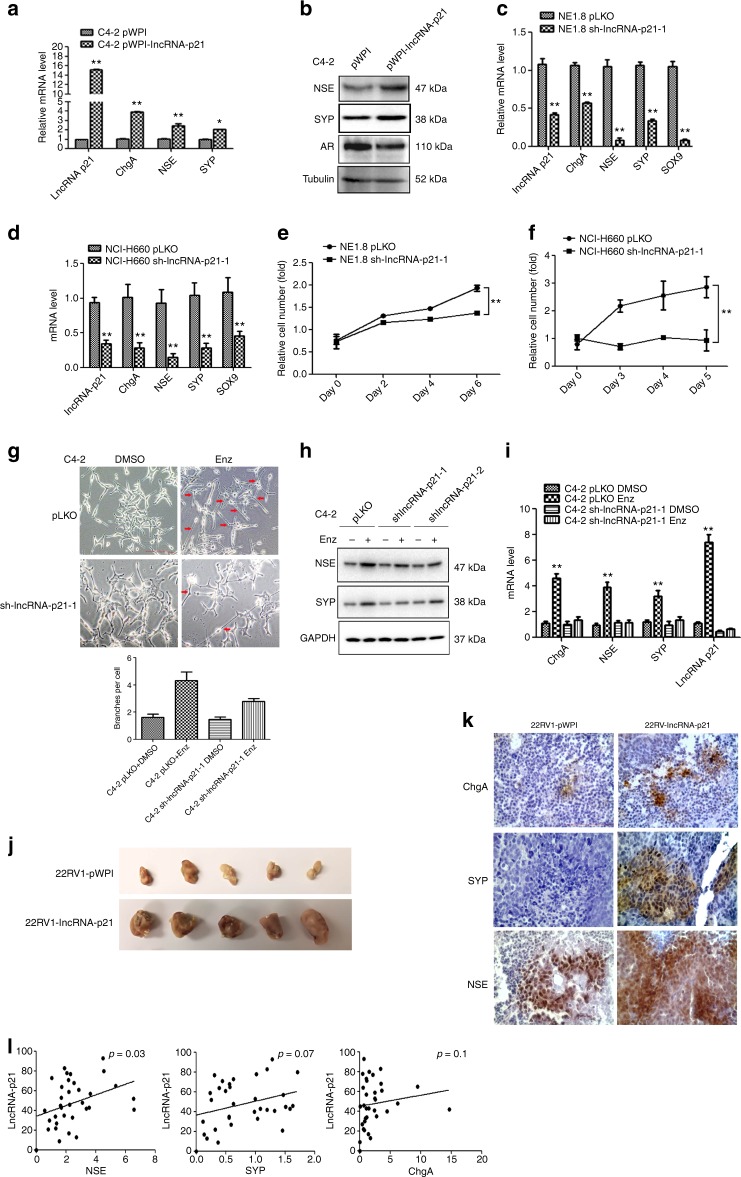
The lncRNA-p21 is the key factor to promote the NED after Enz treatment and maintain the NE cell characteristics. **a** Overexpressing lncRNA-p21 in C4-2 cells for NE markers analysis by qPCR. **b** Overexpressing lncRNA-p21 in C4-2 cells for NE markers analysis by WB. **c** The NE markers in NE1.8 cells after knocking down lncRNA-p21 were determined by qPCR. **d** The NE markers in NCI-H660 cells after knocking down lncRNA-p21 were determined by qPCR. **e** The lncRNA-p21 was knocked down in NE1.8 cells and cell viability was analyzed by MTT. **f** The lncRNA-p21 was knocked down in NCI-H660 cells and cell viability was analyzed by MTT. **g** Cell morphology of pLKO and sh-lncRNA-p21 C4-2 cells after Enz treatment. (Scale bar = 20 μm) **h** Knockdown of lncRNA-p21 decreased the Enz-induced NE marker protein levels. The C4-2 pLKO control and sh-lncRNA-p21 cells were treated with Enz for 6 days, and then the NE markers analyzed by WB. **i** Knockdown of lncRNA-p21 decreased the Enz-induced mRNA of NE markers. The C4-2 pLKO control and sh-lncRNA-p21 cells were treated with Enz for 6 days, and then the NE markers determined by qPCR. **j** Images of 22RV1-pWPI and 22RV1-lncRNA-p21 xenograft tumors. **k** The IHC staining of the NE markers in 22RV1-pWPI and 22RV1-lncRNA-p21 xenograft tumors. **l** The correlation of expressions of LncRNA-p21 and NE markers (NSE, SYP, ChgA) in human prostate cancer samples. (*n* = 34). For **c**–**f**, **i**, **l**, the data are presented as mean ± SD, **p < 0.005. by *t-*test for two groups or ANOVA for more than two groups

To further link the Enz-increased lncRNA-p21 expression to the Enz-enhanced NED, we overexpressed the lncRNA-p21 in PCa cells, and results from the Western Blot (WB) and qPCR assays revealed that adding lncRNA-p21 led to increase the expression of NE markers in both C4-2 and CWR22RV1 cells (Fig. 2a, b and Supplementary Fig. 1C, respectively), and knocking down lncRNA-p21 led to decrease the NE markers expression (Fig. 2c, d and Supplementary Fig. 1F–H) and suppress the cell growth in NE1.8, NCI-H660 and DU145 cells (Fig. 2e, f and Supplementary Fig. 1E and I, respectively), suggesting that lncRNA-p21 is the key player to promote the NED and maintain the NE cell characteristics.

Importantly, knocking down the lncRNA-p21 also blocked the Enz-induced NE-like cellular morphology in C4-2 cells (Fig. 2g), as well as suppressed the Enz-induced NE marker expressions (Fig. 2h, i and Supplementary Fig. 1J).

We also knocked down HNRP-K (a co-factor of lncRNA-p21) and HUR (a suppressor for lncRNA-p21) in C4-2 cells (Supplementary Fig. 1K)^16,35^, and results revealed that Enz treatment failed to enhance NED after depleting HNRP-K (Supplementary Fig. 1K). In contrast, Enz can better promote the NED after knocking down HUR (Supplementary Fig. 1K), suggesting that lncRNA-p21 is the key player for the Enz-induced NED in PCa cells.

To confirm the conclusions from the in vitro cell line studies, we established the CWR22RV1 xenograft tumors, and the results showed that the lncRNA-p21 can enhance the tumor sizes and weights (Fig. 2j and Supplementary Fig. 2A–B). More importantly, NE markers expressions in the CWR22RV1-lncRNA-p21 tumors were significantly increased compared to the CWR22RV1-pWPI tumors (Fig. 2j, k). The further analysis of the levels of lncRNA-p21 and different NE markers (NSE, ChgA and SYP) in human PCa samples also revealed the positive correlations between the expression of lncRNA-p21 and NSE, ChgA, and SYP (Fig. 2l).

Together, results from Fig. 2a–l, Supplementary Fig. 1B–K, and Supplementary Fig. 2 suggest that lncRNA-p21 is essential for the Enz-induced NED.

### EZH2 is essential for Enz-induced NED in PCa

To dissect the molecular mechanism of how Enz induces lncRNA-p21 to promote NED in PCa, we focused on investigating the EZH2, the key player of the PRC2 complex, since a recent study suggested that lncRNA-p21 could regulate PRC2 target genes expression^17^ and EZH2 was found to play key roles for NEPC progression^25^.

**Fig. 3:**
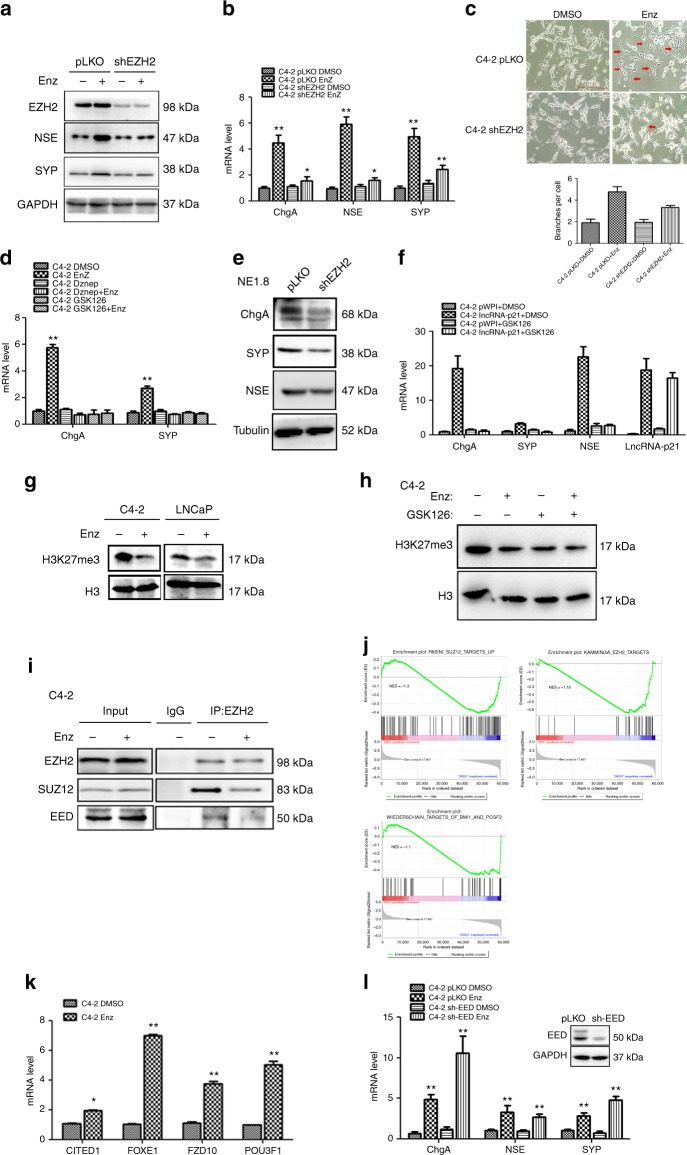
EZH2, but not the PRC2 complex, is essential for Enz treatment induced NED. **a**, **b** Enz-increased NE markers were reduced after EZH2 knockdown. The C4-2 pLKO control and shEZH2 cells were treated with/without (w/o) 10 μM Enz for 4 days, and the NE markers detected by (**a**) western blot (WB) and (**b**) qPCR. **c** EZH2 knockdown can reverse the Enz-induced NED. The C4-2 pLKO and shEZH2 cells were treated w/o Enz for 7 days, then the cell morphology was observed under microscope, quantitations in lower panel. (Scale bar = 20 μm). **d** EZH2 inhibitors can inhibit the expression of NE markers. C4-2 cells were treated w/o Enz and EZH2 inhibitors (4 μM GSK126 and 1 μM Dznep) for 4 days, and the mRNAs of NE markers analyzed by qPCR. **e** Expression levels of NE markers in NE1.8 cells after knocking down EZH2. **f** C4-2 pWPI and pWPI-lncRNA-p21 cells were treated w/o 4 μM GSK126, and the NE markers levels detected by qPCR. **g** H3K27me3 levels in C4-2 cells after treating w/o Enz for 4 days. **h** H3K27me3 levels in C4-2 cells after DMSO, Enz and GSK126 treatment. **i** Co-IP assay to detect the interaction between EZH2, SUZ12 and EED in C4-2 cells w/o Enz treatment for 4 days. **j** GSEA of PRC2 and EZH2 pathway enrichment in C4-2 cells after Enz treatment. **k** Expression of PRC2 complex target genes in C4-2 cells after 4 days of Enz treatment. **l** The mRNA levels of NE markers in C4-2 pLKO control and C4-2 shEED cells after Enz treatment for 4 days. For **b**, **d**, **f**, **k**, the data are presented as mean ± SD, *p < 0.05, **p < 0.005, by *t-*test

We first knocked down EZH2 in C4-2 cells and found that this significantly reduced the Enz-increased NE marker expressions (Fig. 3a, b). The microscopic images of the C4-2 cells also indicated that suppressing the EZH2 resulted in less NE-like structure (Fig. 3c). Importantly, by analyzing the AR and PSA expressions, we found that shEZH2 failed to change the AR activity and the suppression effect of Enz on AR signals (Supplementary Fig. 3A–B), suggesting that EZH2 regulating NED does not function through targeting the AR signals.

Similar results were also obtained when we applied EZH2 specific inhibitors Dznep and GSK126. We found suppressing the EZH2 activity would also block the Enz-induced NED, suggesting that EZH2 methyltransferase activity is required for the Enz-induced NED (Fig. 3d and Supplementary Fig. 3C). Using the NEPC cell line, NE1.8^36^, we obtained similar results showing knocking down EZH2 decreased the NE markers expressions (Fig. 3e). More importantly, treating the NCI-H660 cells with EZH2 inhibitors can significantly suppress the cell growth, which suggested that targeting EZH2 can reduce the NEPC cell viability (Supplementary Fig. 3D).

We further found that treatment with GSK126 abolished the lncRNA-p21-enhanced NED (Fig. 3f), suggesting that the lncRNA-p21-induced NED is dependent on EZH2, and demonstrating that Enz may function via altering lncRNA-p21 to modulate the EZH2-induced NED.

Together, results from Fig. 3a–f and Supplementary Fig. 3C–D suggest that the Enz-induced lncRNA-p21 promotion of NED is dependent on EZH2.

### Enz promotes NED without involving the PRC2 complex

As the prevalent function of EZH2 is to mediate the PRC2 complex function^37^, we were interested to see if EZH2 could alter the Enz-induced NED via altering the PRC2 complex. We found that treating C4-2 cells with Enz significantly decreased the H3K27me3 level, which is the marker for PRC2 complex activity (Fig. 3g, h).

Results from Co-immunoprecipitation (Co-IP) assays also revealed that Enz could dramatically decrease the interaction among 3 PRC2 complex core subunits (EZH2, SUZ12 and EED) in C4-2 and LNCaP cells (Fig. 3i and Supplementary Fig. 3E, respectively), suggesting that Enz can disrupt the formation of the PRC2 complex. Results from the GSEA analysis indicated that Enz increased the expression of PRC2-EZH2 target genes (Fig. 3j and Supplementary Fig. 3F). We also observed that Enz treatment increased several PRC2 target genes expressions in C4-2 cells (Fig. 3k).

To identify whether the PRC2 complex is required for Enz-induced NED, we knocked down other PRC2 complex components including EED and SUZ12 and assayed their impact on the NED. The qPCR results indicated that disruption of the PRC2 complex failed to block the Enz-induced NED in C4-2 cells (Fig. 3l and Supplementary Fig. 3G), suggesting that Enz-induced NED is not dependent on PRC2 complex function.

Together, results from Fig. 3g–l and Supplementary Fig. 3E–G suggest that Enz can increase NED via EZH2, but independent of the PRC2 complex.

### Enz enhances STAT3 methylation via EZH2 to promote the NED

**Fig. 4:**
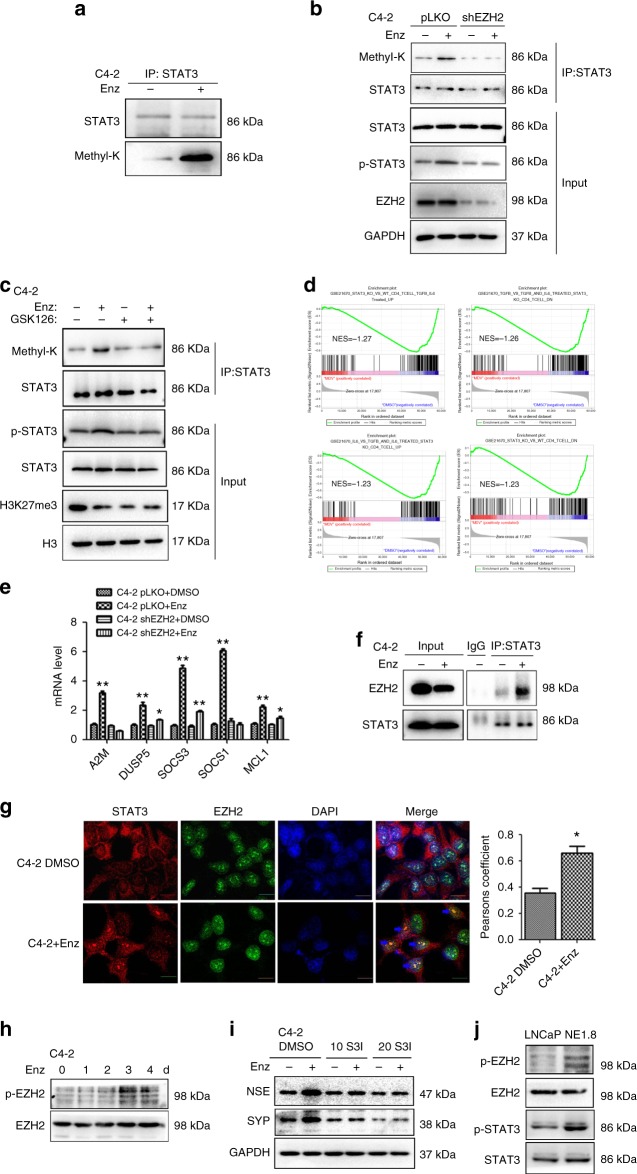
EZH2 methylates STAT3 to promote NED after Enz treatment. **a** STAT3 methylation (Methyl-K) is increased in C4-2 cells treated with/without (w/o) 10 μM Enz for 4 days and the lysine methylation of STAT3 detected by anti-lysine methylation antibody. **b** EZH2 knockdown can reduce Enz increased STAT3 methylation. C4-2 pLKO vector and shEZH2 cells were treated w/o Enz for 5 days, and then STAT3 methylation and phosphorylation detected by WB. **c** EZH2 inhibitor can reduce Enz-Increased STAT3 methylation. The levels of STAT3 methylation and phosphorylation w/o Enz and GSK126 treatment were detected by WB. **d** GSEA of STAT3 pathway enrichment in C4-2 cells with Enz treatment. **e** STAT3 downstream genes’ expressions in C4-2 PLKO and shEZH2 cells after Enz treatment. **f** Co-IP assay to detect the interaction between EZH2 and STAT3 after Enz treatment. C4-2 cells were treated w/o Enz for 5 days. EZH2 antibody was used to pull-down the EZH2 complex, and the STAT3 level detected by WB. **g** Confocal microscopic images of co-localization of EZH2 and STAT3 after Enz or DMSO treatment for 4 days. The co-localization of STAT3 and EZH2 was analyzed by Pearsons coefficient (Scale bar = 2 μm). Quantitation on the right. **h** WB analysis of EZH2 p-S21 in C4-2 cells w/o Enz treatment daily for 4 days. **i** STAT3 inhibitor, S3I-201 (S3I), can reduce the expression of Enz-induced NE markers. C4-2 cells were treated w/o Enz and S3I for 6 days, and the NE markers determined by WB. **j** The p-EZH2, EZH2, p-STAT3 and STAT3 levels in NE1.8 and LNCaP cells were analyzed by WB. For **e**, **g**, the data are presented as mean ± SD, *p < 0.05, **p < 0.005 by *t-*test

To investigate how Enz functions via EZH2 to induce the NED independent of the PRC2 complex, we examined if Enz-induced NED is via altering the EZH2 non-histone methyltransferase function. We first assayed the lysine methylation (Methyl-K) of three proteins that have been reported as EZH2 targets, including STAT3, AR and RORα, and found only methylation of STAT3 can be induced in C4-2 cells after Enz treatment (Fig. 4a and Supplementary Fig. 4A). This result is consistent with early study results indicating that STAT3 could promote the NED^38^.

We then knocked down EZH2 in C4-2 cells, and found that suppressing EZH2 reduced the STAT3 methylation induced by Enz (Fig. 4b). In agreement with the earlier report showing that the methylation of STAT3 enhanced the STAT3 activity^22^, we also found that knocking down EZH2 reduced phosphorylation of STAT3 (Fig. 4b). Treating with GSK126 also significantly reversed the Enz-induced STAT3 methylation, and consequently reduced STAT3 phosphorylation in C4-2 cells (Fig. 4c).

Results from the RNAseq assay also demonstrated that Enz treatment increased STAT3 downstream genes expressions (Supplementary Fig. 4B), which were confirmed from GSEA showing Enz could activate STAT3 signaling (Fig. 4d). As expected, knocking down EZH2 reversed the Enz-increased STAT3 downstream genes expression, suggesting that Enz alters the EZH2 activity to modulate the STAT3 activity in C4-2 cells (Fig. 4e).

The Co-IP assay revealed that Enz treatment could enhance the interaction between EZH2 and STAT3, suggesting that Enz might increase STAT3 methylation via promoting this interaction (Fig. 4f). In image analysis using confocal microscope, we also observed that Enz promotes the translocation of STAT3 from the cytosol into the nucleus to co-localize with EZH2 (Fig. 4g).

Furthermore, we found that Enz enhanced the EZH2 phosphorylation at serine 21 (S21) (Fig. 4h), which is in agreement with the early report that EZH2 S21 phosphorylation by AKT can trigger the EZH2 to methylate STAT3^22^.

Finally, adding either the specific inhibitor S3I-201 or STAT3-shRNA to target the STAT3, we found that suppressing the STAT3 could significantly reduce the Enz-induced NED in C4-2 (Fig. 4i and Supplementary Fig. 4C, respectively). We also found that p-EZH2 and p-STAT3 levels were elevated in NE1.8 cells compared to LNCaP cells, indicating that the EZH2-STAT3 signal is activated in these cells (Fig. 4j).

Together, results from multiple approaches shown in Fig. 4a–j and Supplementary 3A–C suggest that Enz can function via promoting the EZH2 methyltransferase activity to methylate STAT3 to induce the NED in the PCa cells.

### LncRNA-p21 promotes EZH2 to enhance STAT3 methylation

**Fig. 5:**
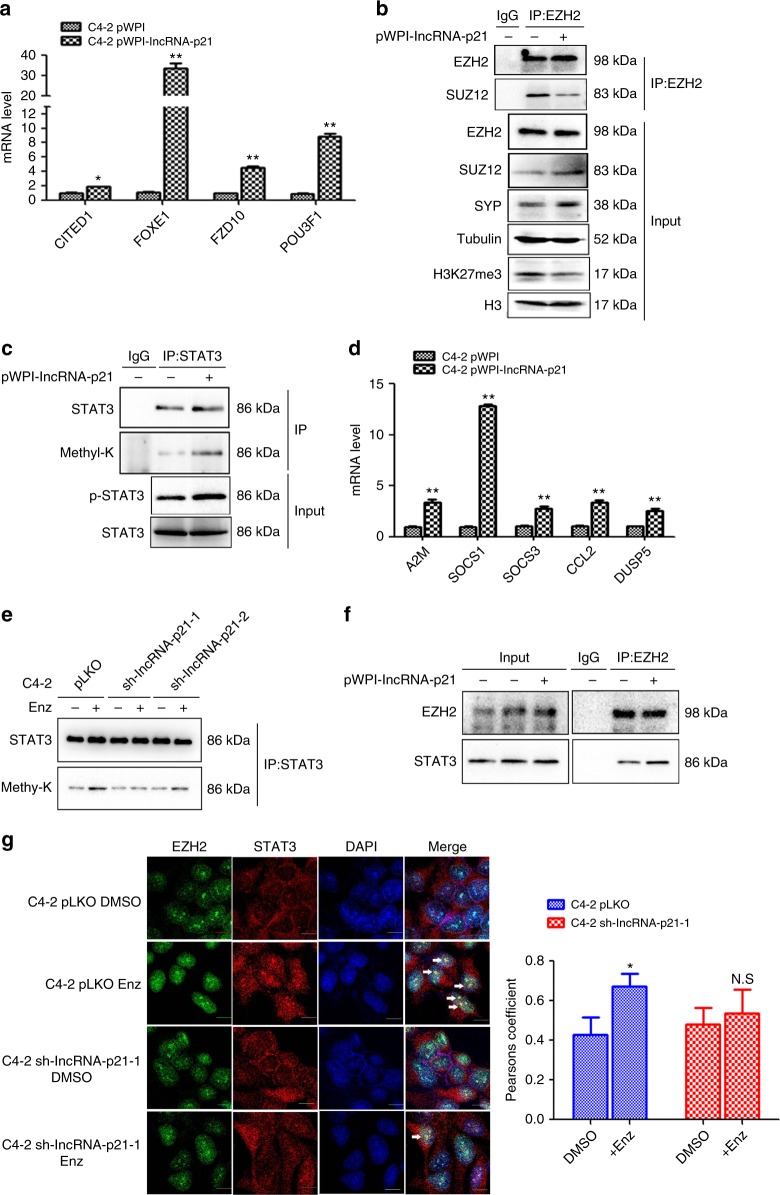
The lncRNA-p21 disrupts the PRC2 complex and promotes EZH2 to methylate STAT3. **a** The qPCR analysis of PRC2 target gene expressions in C4-2 cells after overexpression of lncRNA-p21. **b** EZH2 protein was immunoprecipitated in C4-2 pWPI and pWPI-lncRNA-p21 cells. The co-immunoprecipitated SUZ12 levels were detected by WB. The SYP and H3K27Me3 levels in input samples were also detected by WB. **c** The WB analysis of STAT3 methylation and phosphorylation with/without (w/o) overexpression of lncRNA-p21 in C4-2 cells. **d** The qPCR analysis of STAT3 target genes expressions in C4-2 cells w/o overexpression of lncRNA-p21. **e** The lncRNA-p21 knockdown can reduce Enz-induced STAT3 methylation. C4-2 pLKO and 2 different sh-lncRNA-p21 transfected cells were treated w/o Enz for 4 days. The STAT3 methylation was detected by WB. **f** Co-IP assay to detect the interaction between EZH2 and STAT3 w/o overexpression of lncRNA-p21 in C4-2 cells. **g** Confocal microscopic images of co-localization of EZH2 and STAT3 w/o Enz treatment in C4-2 pLKO and sh-lncRNA-p21 cells. The co-localization of EZH2 and STAT3 was analyzed by Pearsons coefficient (Scale bar = 2 μm). Quantitation on the right. For **a**, **d**, **g**, data are presented as mean ± SD, *p < 0.05, **p < 0.005, N.S. not significant by *t-*test

To investigate how Enz induces lncRNA-p21 to regulate the EZH2 function, we checked whether lncRNA-p21 can regulate the PRC2 complex activity. We found adding lncRNA-p21 increased the expression of several EZH2-PRC2 target genes and suppressed the interaction between EZH2 and other PRC2 complex components and reduced the H3K27me3 (Fig. 5a, b). In contrast, knocking down lncRNA-p21 increased EZH2 and SUZ12 interaction (Supplementary Fig. 5A).

Importantly, we found adding lncRNA-p21 in C4-2 cells also significantly enhanced the STAT3 methylation and phosphorylation (Fig. 5c and Supplementary Fig. 5B), as well as the STAT3 downstream genes expression (Fig. 5d). As expected, knocking down lncRNA-p21 blocked the Enz-induced STAT3 methylation (Fig. 5e and Supplementary Fig. 5C), suggesting that Enz may function via increasing the lncRNA-p21 to alter the EZH2 methyltransferase activity to enhance the STAT3 methylation.

The results from Co-IP assays also revealed that lncRNA-p21 could promote the interaction of EZH2 with STAT3 (Fig. 5f), and knocking down the lncRNA-p21 in the C4-2 cells also rendered the Enz to have less effect to promote the STAT3 and EZH2 co-localization (Fig. 5g), suggesting that lncRNA-p21 is necessary for the Enz-induced STAT3 and EZH2 interaction.

Together, results from Fig. 5a–g suggest that Enz promotes the lncRNA-p21 expression to increase the EZH2 non-histone methyltransferase activity to methylate STAT3, which then results in NED induction in PCa.

### LncRNA-p21 inhibits EZH2 and Hotair interaction

**Fig. 6:**
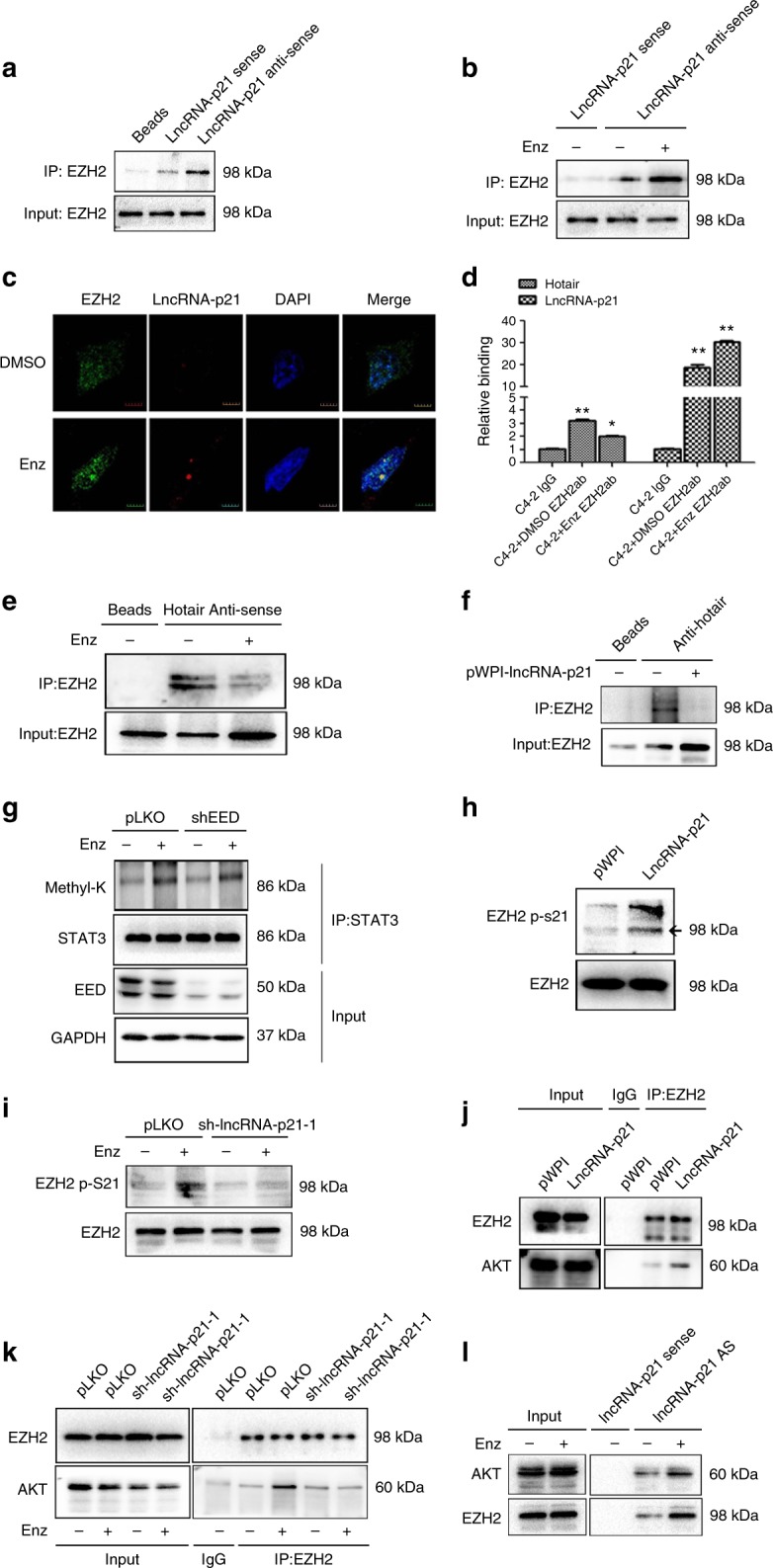
The lncRNA-p21 competes with Hotair to interact with EZH2 and promotes phosphorylation of EZH2 at S21. **a**, **b** RNA-IP was performed to detect the lncRNA-p21 interaction with EZH2 by using lncRNA-p21 anti-sense probe vs sense probe in (**a**) C4-2 cells and in (**b**) Enz-treated C4-2 cells. **c** RNA in situ hybridization and immunofluorescent staining to detect the co-localization of lncRNA-p21 and EZH2 in C4-2 cells w/o Enz treatment (Scale bar = 2 μm). **d** In C4-2 cells, the EZH2 was pulled down by EZH2 antibody and the levels of lncRNA-p21 and Hotair were detected by qPCR. **e**, **f** RNA-IP assay to detect the interaction between Hotair and EZH2 after treating w/o Enz (**e**) and w/o overexpression of lncRNA-p21. **f**, **g** STAT3 methylation levels were detected by WB in C4-2 pLKO and sh-EED cells after 4 days w/o Enz treatment. **h** EZH2 p-S21 levels were detected in C4-2 PWPI and PWPI-lncRNA-p21 cells. **i** EZH2 p-S21 level was detected in C4-2 pLKO and sh-lncRNA-p21 cells w/o Enz treatment. **j** The Co-IP assay to detect the AKT and EZH2 interaction in C4-2 pWPI and C4-2 pWPI-lncRNA-p21 cells. **k** C4-2 pLKO and sh-lncRNA-p21 cells were treated w/o Enz for 4 days and then EZH2 was immunoprecipitated. The AKT and EZH2 interaction was analyzed by WB. **l** C4-2 cells were treated w/o Enz, and then the lncRNA-p21 was pulled down. The interaction of AKT and EZH2 with lncRNA-p21 was analyzed by WB. For **d**, the data are presented as mean ± SD, *p < 0.05, **p < 0.005, by ANOVA

To further study how lncRNA-p21 disrupts the PRC2 complex, we examined whether it can directly interact with EZH2. Results from RNA pull-down assays indicated that lncRNA-p21 could bind to EZH2, and Enz treatment significantly increased this binding (Fig. 6a–c).

Results from the RIP assay also demonstrated the interaction between lncRNA-p21 and EZH2 and Enz treatment could enhance lncRNA-p21 binding to EZH2 in C4-2 cells (Fig. 6d). Interestingly, we also found that Enz significantly suppressed the interaction of EZH2 and lncRNA-Hotair, the key factor to stabilize the PRC2 complex^39^ (Fig. 6d). Similar results were also obtained in RNA pull-down assays, demonstrating that Enz reduced lncRNA-Hotair and EZH2 interaction (Fig. 6e), and adding lncRNA-p21 could then suppress EZH2 and lncRNA-Hotair interaction (Fig. 6f).

Together, results from Fig. 6a–f suggest that Enz increases the lncRNA-p21 level to disrupt the PRC2 complex and this may be achieved through inhibiting the Hotair and EZH2 interaction. The consequences of such inhibition result in releasing the EZH2 from the PRC2 complex and increasing the EZH2 non-histone methyltransferase activity to methylate the STAT3.

### LncRNA-p21 enhances EZH2 p-S21 to increase STAT3 methylation

To dissect the mechanism how the Enz induced lncRNA-p21 promotes the released EZH2 to methylate STAT3, we examined whether simply disrupting the PRC2 complex can result in STAT3 methylation induction. Our results indicated that knocking down EED, the key component of the PRC2 complex^21^, failed to alter the STAT3 methylation status (Fig. 6g), yet Enz could still increase the STAT3 methylation in the EED-shRNA cells, suggesting that only disruption of the PRC2 complex is not adequate for the Enz-enhanced STAT3 methylation. To further investigate how Enz-lncRNA-p21 promotes STAT3 methylation, we raised two hypotheses, (1) lncRNA-p21 functions as a scaffold to interact with EZH2 and STAT3, or (2) lncRNA-p21 could promote the EZH2 p-S21 level to enhance the STAT3 methylation. Results from the interaction of lncRNA-p21, EZH2, and STAT3 molecules revealed that Enz treatment increased the lncRNA-p21 interaction with EZH2, but not STAT3 and EED (Supplementary Fig. 6), suggesting that lncRNA-p21 may not be able to function as the scaffold for EZH2 and STAT3.

We then examined the effect of Enz-enhanced EZH2 p-S21 level and found lncRNA-p21 increased EZH2 p-S21 (Fig. 6h), and knocking down lncRNA-p21 significantly blocked the Enz-induced EZH2 p-S21 (Fig. 6i).

Since an early report indicated that AKT could phosphorylate EZH2 S21 and promote the STAT3 methylation^22^, we also studied AKT roles. It was found that the lncRNA-p21 dramatically increased EZH2 and AKT interaction, and sh-lncRNA-p21 blocked the Enz-increased interaction between EZH2 and AKT (Fig. 6j, k). Importantly, results from the interaction between lncRNA-p21, EZH2, and AKT indicated that these molecules can interact with each other, and the Enz treatment enhances such an interaction (Fig. 6l). These results suggest that the lncRNA-p21 could function as a scaffold to facilitate the EZH2 and AKT interaction.

Together, results from Fig. 6g–l suggest that Enz-lncRNA-p21 can promote AKT-EZH2 interaction to increase the EZH2 p-S21 level. The consequence of these two regulation steps (disruption of the PRC2 complex to release the EZH2 and enhancement of the AKT and EZH2 interaction), in turn, increased the STAT3 methylation.

### Enz differentially controls AR binding to lncRNA-p21 promoter

All above results suggest that ADT-Enz can promote NED via altering the lncRNA-p21/EZH2/STAT3 axis. To further dissect the mechanism(s) of how Enz can increase lncRNA-p21 expression, we investigated the role of AR, since ADT-Enz was developed mainly to target the AR signals. As shown in Supplementary Fig. 7A, Enz can not induce the lncRNA-p21 expression in C4-2 shAR cells, suggesting that AR is the critical factor for the Enz-induced lncRNA-p21 expression.

**Fig. 7:**
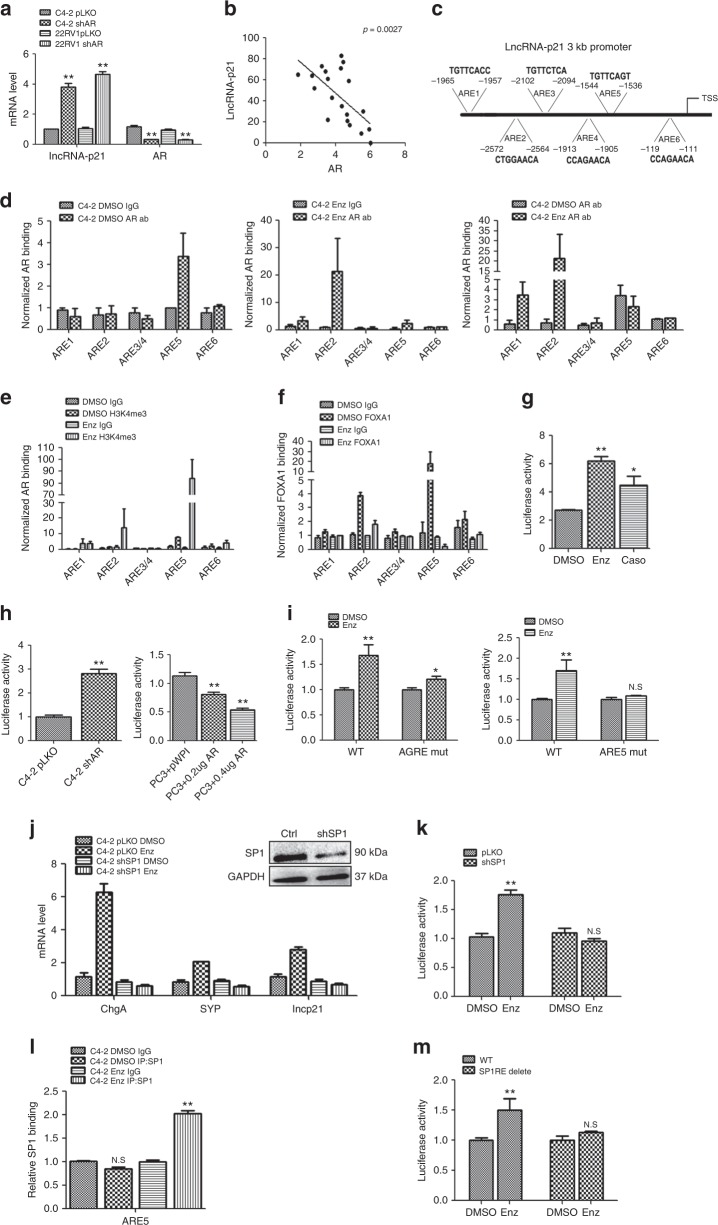
Enz differentially regulates AR binding on the lncRNA-p21 promoter region and promotes lncRNA-p21 expression. **a** The qPCR analysis of lncRNA-p21 expression in C4-2 and CWR22RV1 (22RV1) cells w/wo AR knockdown. **b** The correlation of AR and lncRNA-p21 expressions in the human PCa samples (*n* = 20). **c** The schematic depiction of putative androgen response elements (AREs) on lncRNA-p21 3 kb promoter region. **d** ChIP assays to identify the AR binding in the putative AREs w/wo Enz treatment. The C4-2 cells were treated w/o Enz for 3 days, and then the AR binding to the putative AREs on the lncRNA-p21 promoter was analyzed by qPCR. **e**, **f** ChIP assays to identify (**e**) H3K4me3 enrichment and (**f**) FOXA1 binding to the putative ARE regions on the lncRNA-p21 promoter. **g** Luciferase assay to identify the transactivation of lncRNA-p21 3 kb promoter region after different anti-androgen treatments. **h** Luciferase assay to identify the transactivation of lncRNA-p21 3 kb promoter region in C4-2 cells with pLKO or shAR (left) and in PC3 cells with pWPI or OE-AR (right). (*p < 0.05, **p < 0.005). **i** The Enz treatment vs DMSO control effects on mutated AGRE (AGRE-mut) and mutated ARE5 (ARE5-mut) vs wild type (WT) lncRNA-p21 promoter transactivation were analyzed by luciferase assay in C4-2 cells. **j** The qPCR to detect the levelS of lncRNA-p21 and NE markers in C4-2 pLKO and shSP1 cells w/o Enz treatment. **k** C4-2 pLKO and shSP1 cells were treated w/o Enz, and then the lncRNA-p21 promoter transactivation activity was analyzed by luciferase assay. **l** ChIP assay to identify the SP1 binding on the ARE5 region before and after Enz treatment. **m** The SP1RE cluster on lncRNA-p21 promoter region was deleted and constructed into pGL3 luciferase reporter plasmid. Enz effects on WT-lncRNA-p21 promoter or SP1RE deleted-lncRNA-p21 promoter transactivation were analyzed by luciferase assay. For **i**, **k**, the data are presented as mean ± SD, *p < 0.05, **p < 0.005, N.S. not significant by *t* test for two groups or ANOVA for more than two groups

The results from the qPCR assay revealed that knockdown of AR significantly increased the lncRNA-p21 expression in both C4-2 and CWR22RV1 cells (Fig. 7a). In contrast, overexpression of AR in PC3 and DU145 cells can dramatically reduce lncRNA-p21 expression (Supplementary Fig. 7B), and treating with the androgen, DHT, can suppress lncRNA-p21 expression (Supplementary Fig. 7C), suggesting that lncRNA-p21 expression is directly regulated by AR. The clinical survey also showed that the AR and lncRNA-p21 expressions are negatively correlated with each other (Fig. 7b). As expected, knocking down AR also significantly increased STAT3 methylation and decreased the interaction between EZH2 and SUZ12, which is consistent with our previous data (Supplementary Fig. 7D–E).

To further dissect the mechanism of how Enz can regulate the lncRNA-p21 expression in PCa cells, we searched for the androgen-response-elements (AREs) on the lncRNA-p21 promoter region, and found 6 putative AREs on the 3 Kb promoter regions (Fig. 7c). The results from the ChIP assays indicated AR could only bind to the ARE5 without Enz treatment (Fig. 7d). However, it was found that treating PCa cells with Enz decreased the AR binding to ARE5 yet surprisingly increased the AR binding to the ARE1 and ARE2 (Fig. 7d).

In addition to the classic AREs, recent reports suggested that Enz could also drive AR to bind to the different response elements, (named as AR antagonist response element, AGRE), with sequence 5′-NCHKGNnndDCHDGN-3′)^40^. Interestingly, we found such an AGRE (5′-TCTTGGTTTGCCTGG-3′) located 27 bp upstream of ARE2, and results from the ChIP sequencing online database indicated that Enz (and Casodex, another antiandrogen) could increase the AR binding on the AGRE region (Supplementary Fig. 7F).

To identify which AREs or AGRE can mediate the Enz-enhanced lncRNA-p21 transcription, we examined the H3K4me3 status around all of the putative AREs and the AGRE, and results revealed that the H3K4me3 status on both AGRE and ARE5 areas was increased significantly after Enz treatment (Fig. 7e), suggesting that the genes transcription on these two areas are active^41^.

Importantly, we also detected the FOXA1 binding on these 2 areas since FOXA1 is the key factor to facilitate the AR binding to DNA^42^. The results from the anti-FOXA1 ChIP assay indicated that only the ARE2 and ARE5 regions showed significant FOXA1 binding (Fig. 7f). We further found that treating C4-2 cells with Enz significantly suppressed the binding of FOXA1 on the ARE5 region. However, Enz treatment only resulted in some decreases of FOXA1 binding to the ARE2 region (Fig. 7f). These results suggest that Enz may drive AR to bind to the AGRE site.

Next, we constructed the 3 kb lncRNA-p21 promoter region to the PGL3 luciferase reporter plasmid to test whether ADT-Enz can increase the lncRNA-p21 transcription. The results from the luciferase assay revealed that Enz (and Casodex) treatment could increase lncRNA-p21 promoter activity, with Enz showing a more significant effect (Fig. 7g). As expected, treating with DHT led to significantly decreased lncRNA-p21 promoter activity and further treating with Enz then partially reversed such DHT-mediated inhibition (Supplementary Fig. 7G).

Similar results were also obtained when we replaced Enz with AR-cDNA/AR-shRNA. Adding the AR-shRNA increased the lncRNA-p21 promoter activity and adding the AR-cDNA decreased the promoter activity (Fig. 7h). Importantly, in AR-shRNA cells, Enz and Casodex treatment lost their ability to increase the lncRNA-p21 promoter activity (Supplementary Fig. 7H). These results suggested that AR plays the suppressor role on the lncRNA-p21 transcription without Enz treatment.

We also constructed different mutants of lncRNA-p21 AREs or AGRE into the PGL3 plasmid, and results revealed that Enz can only slightly increase the lncRNA-p21 promoter activity with mutated AGRE. Similar to AGRE, Enz had less ability to increase the lncRNA-p21 promoter activity with mutated ARE5 (Fig. 7i), suggesting that Enz blocked the AR binding to ARE5 and increased the lncRNA-p21 transcription, and Enz has a unique capacity to promote the AR binding to AGRE and further promote the lncRNA-p21 expression.

Together, results from Fig. 7a–i suggest that AR may play a suppressor role to inhibit lncRNA-p21 expression when binding to the ARE5, while play a promoter role to activate lncRNA-p21 expression when binding to the AGRE.

Further mechanism dissection with sequence analysis found that there is a cluster of SP1 binding sites close to ARE5 (Supplementary Fig. 7I). SP1 is a transcription factor that can drive various genes expression^43^. Since SP1 binding sites are close to ARE5, we were interested to see if AR binding to ARE5 may suppress the SP1 binding to its binding sites, and treating with Enz may release AR binding to promote the SP1 binding. As expected, knocking down SP1 significantly attenuated lncRNA-p21 and NE markers induction after Enz treatment (Fig. 7j). The luciferase assay also indicated that in sh-SP1 cells, Enz treatment failed to increase lncRNA-p21 promoter activity (Fig. 7k). The results from the ChIP assay revealed that both Enz treatment and charcoal dextran treated (CD) media could enhance SP1 binding on the ARE5 region (Fig. 7l and Supplementary Fig. 7J, respectively). The mutation of the SP1 binding site resulted in Enz losing most of its ability to increase the lncRNA-p21 transcription (Fig. 7m).

Together, results from Fig. 7i–m suggest that Enz treatment can enhance SP1 binding on its binding sites near the ARE5 region to promote the lncRNA-p21 transcription.

### EZH2 inhibitor blocks Enz-induced NED in PDX mouse model

**Fig. 8:**
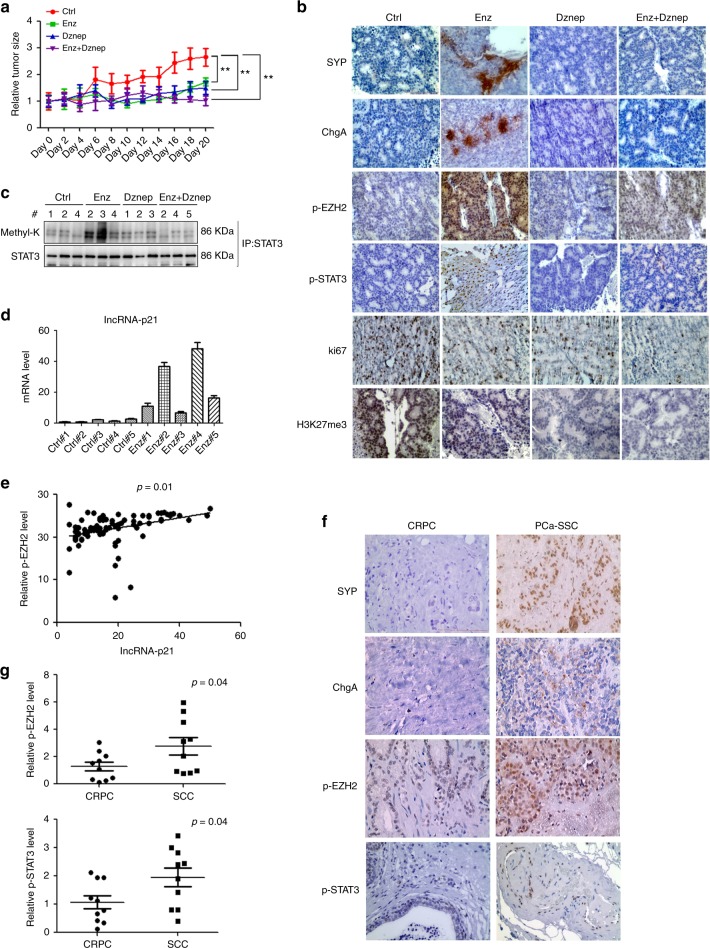
LncRNA-p21/EZH2/STAT3 signal is activated in PDX mice model after Enz treatment and human NEPC samples. **a** The PCa PDX samples were subcutaneously implanted into SCID mice and 5 mice/group received different i.p. treatments (1. DMSO; 2. Enz; 3. Dznep; 4. Dznep + Enz) every other day, and tumor sizes were measured after different treatments. After 10 treatments, the mice were kept for another 2 days and then were sacrificed.The PDX tumors were collected, and the relative tumor growth rates of different treatments were compared. **b** The IHC staining to identify the level of NE markers indicated in different groups (Scale bar = 20 μm). **c** Three tumor samples were randomly picked up from each group, the tissues were lysed and then STAT3 methylation (methyl-k) was detected by WB. **d** The lncRNA-p21 levels in control group and Enz-treated group were detected by qPCR. **e** The correlation of lncRNA-p21 expression and p-EZH2 level in human PCa samples (*n* = 80). **f** Representative images of the immunohistochemistry staining of SYP, ChgA, p-EZH2 and p-STAT3 in human CRPC and SCC samples. (Scale bar = 20 μm). **g** The quantification of p-EZH2 (upper) and p-STAT3 (lower) levels in human CRPC and PCa-SCC samples. For **a**, **e**, **g**, the data are presented as mean ± SD, **p < 0.005, by *t* test for two groups or ANOVA for more than two groups

To confirm all above in vitro cell lines data in the in vivo mouse model, we implanted the PCa patient-derived xenograft (PDX) samples into 20 SCID mice. After the average tumor volume reached 200 mm^3^, we randomly divided mice into 4 groups for i.p injections of the control DMSO, Enz (30 mg/kg) and/or EZH2 inhibitor-Dznep (1 mg/kg) every other day. The results revealed that both Enz and Dznep treatment significantly reduced the PCa growth **(**Fig. 8a, b and Supplementary Fig. 8A**)**. Results from IHC staining of the NE markers (SYP and ChgA) in tumor tissues of Enz-treated mice also indicated that Enz significantly increased the NED (Fig. 8b**)**. However, Dznep treatment reduced Enz-induced SYP and ChgA expressions, suggesting that blocking EZH2 can suppress the Enz-induced NED (Fig. 8b).

Our in vitro study suggests that Enz can block the PRC2 complex. By detecting the H3K27me3 level in the PDX samples, we obtained similar results that Enz treatment significantly decreased the H3K27me3 level in the PDX samples (Fig. 8b). We also confirmed the up-regulation of p-EZH2 and p-STAT3 after Enz treatment in PDX samples (Fig. 8b), and reduction of p-STAT3 level after Dznep treatment (Fig. 8b), suggesting that EZH2 is essential for Enz to increase the STAT3 activity.

To validate that Enz can promote the EZH2 interaction with STAT3 and methylate the STAT3 molecule, we performed the immunofluorescent staining for the EZH2 and STAT3 in Enz-treated PDX samples (vehicle-treated samples were used as control). The results suggested that after Enz treatment, STAT3 can co-localize with EZH2 in nucleus (Supplementary Fig. 8B). Importantly, results from assaying the PDX samples also revealed that Enz could increase the STAT3 methylation, and treating with Dznep could block such induction (Fig. 8c), which is consistent with our in vitro studies showing Enz can function via altering the EZH2 to promote STAT3 methylation.

Finally, results from our qPCR also showed Enz treatment can promote the lncRNA-p21 expression in PDX samples (Fig. 8d) to trigger the EZH2-STAT3 axis.

Together, results from our in vivo PDX mouse studies validate our in vitro cell lines results showing that Enz can promote NED via activating the lncRNA-p21/EZH2/STAT3 axis, so we suggest that targeting this axis with an EZH2 inhibitor may suppress the Enz-induced NED in the PCa.

### EZH2/STAT3 signal is activated in the human NEPC samples

Finally, to confirm the existence of the lncRNA-p21/EZH2/STAT3 axis in the human clinical samples, we investigated the correlation of lncRNA-p21 and p-EZH2. We found that lncRNA-p21 expression is also correlated with the p-EZH2 level in PCa samples (Fig. 8e). Importantly, we detected significantly higher numbers of positively stained cells with p-EZH2 and p-STAT3 antibodies (Fig. 8f, g), suggesting a positive linkage from lncRNA-p21 to EZH2 to STAT3 signaling in human SCC cells that is consistent with our in vitro and in vivo studies.

## Discussion

ADT-Enz treatment, currently used as the final therapeutic strategy to suppress the metastatic CRPC (mCRPC), may extend the patients’ survival an extra 4.8 months^44^. However, the ADT-Enz treatment may also have some unwanted adverse effects, including NED induction. Clinical data indicated that 30–40% mCRPC patients have the NEPC cells, with 10% of them being small cell carcinomas (SCC) and the other 20–30% are NE-like cells^45^, which may gradually progress to the SCC with much higher proliferation rates and metastasis abilities^4^, as well as being resistant to the ADT-antiandrogens treatment since these NEPC cells express little AR^24^, and the average survival rate after detection is less than 1 year^46^.

Mechanism dissection suggested that the development of NEPC may be through multiple mechanisms. For example, 40% of NEPC cells have AURKA and MYCN amplification^24^. The activated AURKA in NEPC may then increase the cell proliferation in the absence of AR, and the MYCN amplification may cooperate with EZH2 to reduce the AR transactivation activity and promote the NED^25^. Other studies also identified that PEG10 might act as an activator to promote the NED in PCa cells^47^, and BRN2 gene was found to be able to promote the NED^5^.

Since the classical ADT has little effect to suppress NEPC, other therapies have been suggested or developed to suppress the NEPC cell growth. For example, a AURKA inhibitor was developed to specifically suppress the NEPC growth without influencing the adenocarcinoma cells growth^24^. Other studies using the GSK503, an EZH2 inhibitor, also suppressed the NED^10^. Our preclinical studies using the EZH2 inhibitors (Dznep and GSK126) also demonstrated that targeting the AR/lncRNA-p21/EZH2/STAT3 axis with EZH2 inhibitors might be able to suppress Enz-induced NED. However, none of these therapeutic strategies have passed human clinical trials.

**Fig. 9:**
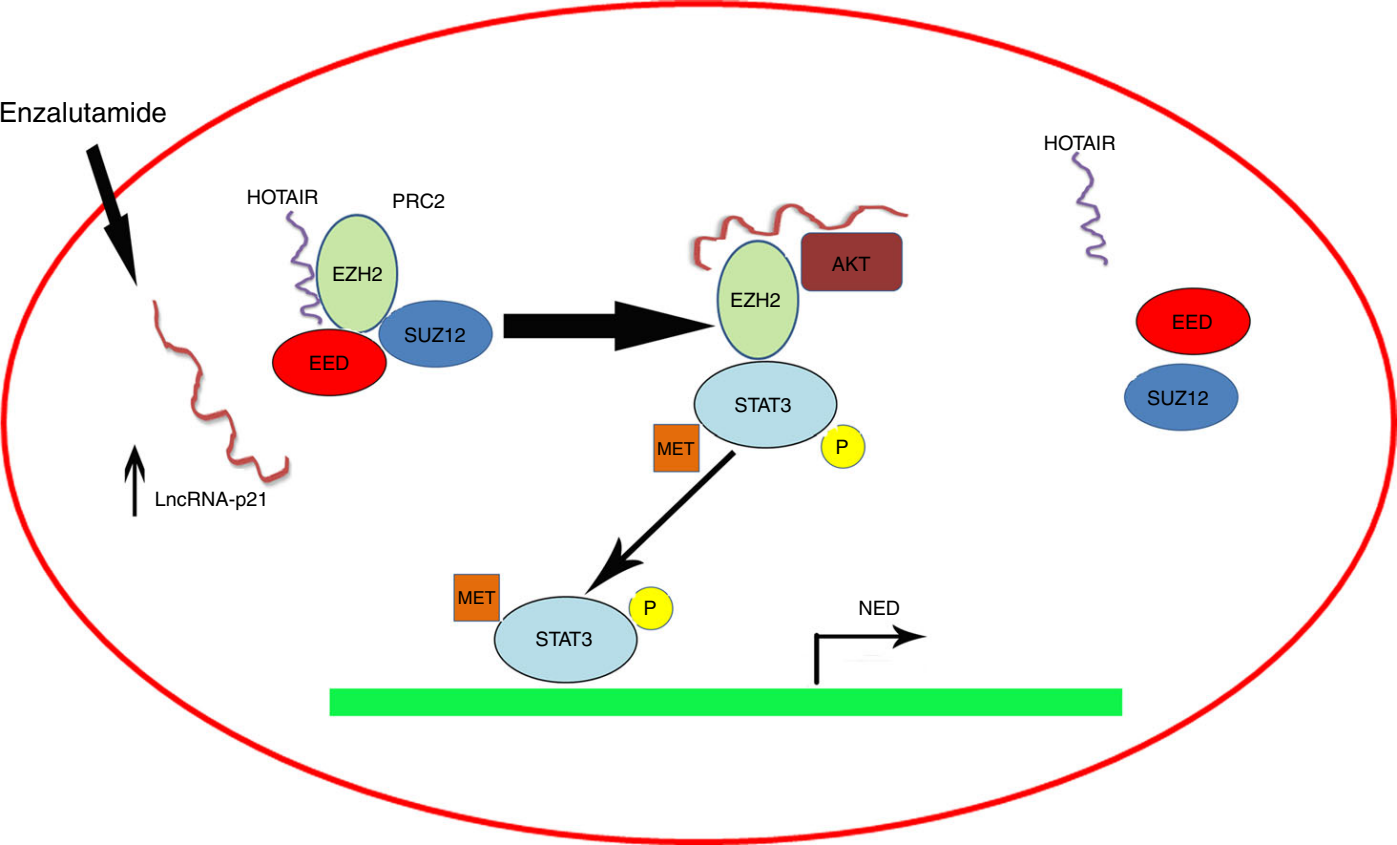
Schematic depiction of lncRNA-p21/EZH2/STAT3 pathway. Enz treatment induces lncRNA-p21 expression via regulating the AR binding to the lncRNA-p21 promoter in PCa cells. Highly expressed lncRNA-p21 competes with Hotair to interact with EZH2 and disrupt PRC2 complex (SUZ12 and EED). At the same time, lncRNA-p21 promotes AKT phosphorylated EZH2 on Serine 21 and enhances the STAT3 methylation by EZH2. The methylated STAT3 was activated and promotes the NED

Our finding that Enz may release EZH2 from the PRC2 complex with increased EZH2 activity that is responsible for the methylation of the STAT3 molecule, which will in turn induce NED increase, is different from previous studies linking the increased EZH2 activity to altered PRC2 function. We suggest that the altered EZH2 activity is independent from the PRC2 function. The discovery of dual functions of Enz, to function via either PRC2 complex dependent or independent manners, is of clinical significance and may help in the development of inhibitors to suppress the NED (Fig. 9 Diagram).

Early studies indicated that STAT3 might play key roles to modulate the PCa progression and their stem cell self-renewal^48–50^, and constitutively activated STAT3 could also impact the Enz-resistance^51^. However, the potential mechanism(s) to activate STAT3 in PCa after the ADT remains unclear. Our results showing Enz may increase the EZH2 activity to methylate STAT3 to increase the NED induction represent the first finding to link the activation of STAT3 (by EZH2) to the Enz-induced NED.

Furthermore, early studies suggested that many lncRNAs might interact with EZH2 and modulate EZH2 PRC2 dependent function^17^. In contrast, our results revealed that lncRNA-p21 might disrupt the PRC2 complex to activate the PRC2 downstream gene expressions. However, lncRNA-p21 not only releases EZH2 from the PRC2 complex, it might also promote the EZH2 interaction with STAT3 to trigger STAT3 methylation, suggesting lncRNA-p21 can control the dual phases of EZH2 function.

In this manuscript, we used multiple different PCa cell lines, C4-2, CWR22RV1, PC3, DU145 and LNCaP, plus two NEPC cell lines, NCI-H660 and NE1.8. Among the cell lines which we used, C4-2 and CWR22RV1 are CRPC cells, LNCaP is an androgen dependent cell line and NE1.8 is a NEPC cell line. The C4-2 and NE1.8 cells are derived from LNCaP cells, but the CWR22RV1 cells are derived from an androgen dependent CWR22 xenograft^52^. Although the C4-2 and CWR22RV1 cells are CRPC cells, much evidence clearly showed that these 2 cell lines are still dependent on AR and inhibition of AR by the anti-androgen (Enz) or shRNA still can suppress the cell growth and promote the NED^2,53–55^. As there is clear evidence suggesting that NEPC cells can be derived from PCa adenocarcinoma cells, we are particularly interested in this transition in response to ADT or suppression of AR signaling. Thus, although the eventual NEPC cells are AR negative, the precursor for NEPC tumors are likely AR positive, and indeed responsive to suppression of AR signaling including Enz treatment, particularly for the cell lines that we used, such as C4-2 and CWR22RV1^53,56,57^.

In summary, our results suggest how Enz treatment can promote NED, and provide some potential therapies of using EZH2 inhibitors to target the AR/lncRNA-p21/EZH2/STAT3 axis to reduce the Enz-induced unwanted adverse effects of promoting the NED.

## Methods

### Cell culture

NCI-H660 (CRL-5813), DU145 (HTB-81), PC-3 (CRL-1435), LNCaP (CRL-1740), 293 (CRL-1573) and CWR22RV1 (CRL-2505) cell lines were purchased from the American Type Culture Collection (ATCC, Manassas, VA). The C4-2 cell line was a gift from Dr. Leland W.K Chung and NE1.8 cell line was a gift from Dr. Ming-Fong Lin. All of the PCa cancer cells were cultured in RPMI-1640 media supplemented with 10% FBS in the humidified 5% CO2 environment at 37 °C. The 293 cells were cultured in DMEM media supplemented with 10% FBS in the humidified 5% CO2 environment at 37 °C. All cell lines were tested to be negative for mycoplasma contamination by direct PCR.

### Lentivirus packaging and infection

The lentivirus system was applied to introduce the shRNAs or overexpression-cDNAs into the PCa cells. The lentiviruses were genrated in 293 cells. The 293 cells were co-transfected with the psAX2 plasmid, pMD2G plasmid and the transfer plasmids. After 48 h transfection, the supernatants which included viruses were collected for immediate use and/or frozen at −80 °C for later use.

### Patient-derived xenograft

The PDX samples were gifts from Dr Sankar N. Maity^29^. The 0.5 cm^3^ xenograft tumor tissues were implanted into the subcutaneous pocket on the severe combined immunodeficient (SCID) mice for amplification of the PDX samples.

### Antibodies and reagents

STAT3 (sc-482), AR (sc-816), GAPDH (sc-47724), tubulin (sc-23948), ChgA (sc-1488), SYP (sc-17750) and ki67 (sc-23900) antibodies were from Santa Cruz Biotechnology, Inc (Santa Cruz, CA). H3K27me3 (#9733), H3K4me3 (#9751), EZH2 (#5246) and p-STAT3 (#9145) antibodies were from Cell Signaling Technology, Inc (Danvers, MA). The AKT (A01486), FOXA1 (EB05999), NSE (AP2780a), H3 (620–360), Methyl-K (SPC-158F), EED (A5371) and SUZ12 (AP20347b) antibodies were from OWL, Inc (San Diego, CA) and p-EZH2 antibody (IHC-00388) from Bethyl Laboratorys, Inc. (Montgomery, TX). RORα (GTX100029) antibody was from GeneTex (Irvine, CA).

### Western blot analysis

Cells were lysed in lysis buffer and proteins (10–30 µg) were separated on 8–10% SDS/PAGE gel and then transferred onto PVDF membranes (Millipore, Billerica, MA). After blocking membranes, they were incubated with primary antibodies, HRP-conjugated secondary antibodies, and visualized using the ECL system (Thermo Fisher Scientific, Rochester, NY). The primary antibodies of STAT3, EZH2, EED, SUZ12, SYP, H3K27me3, H3, p-STAT3, AKT, Methyl-K and NSE were used at dilutions of 1:1000. The primary antibodies of AR, tubulin and GAPDH were used at dilutions of 1:2000. The primary antibody ChgA was used at a dilution of 1:500. Uncropped blots of Figs. 2b, h, 3a, e, g, i, 4b, h, j, 5b, 6b, e, f, h, k and 8c are provided in Supplementary Fig. 9.

### STAT3 methylation assay

The cells were lysed in lysis buffer and then incubated with STAT3 antibody (2 µg) overnight and incubated with 10 µl Agarose A/G beads for 1 h at 4 °C. The methylation level of STAT3 was analyzed by WB using the lysine methylation antibody.

### RNA extraction and quantitative real-time PCR (qPCR) analysis

Total RNAs were isolated by Trizol (Invitrogen, Grand Island, NY). 1 µg of total RNA was subjected to reverse transcription using Superscript III transcriptase (Invitrogen). Quantitative real-time PCR (qRT-PCR) was conducted using a Bio-Rad CFX96 system with SYBR green to detect the mRNA expression level of a gene of interest. Expression levels were normalized to the expression of Tubulin or GAPDH. The primers used for the genes of interest are listed in Supplementary Data 2.

### Cell growth assay

The DU145, NE1.8 and NCI-H660 cells were infected with pLKO or sh-lncRNA-p21 viruses. And then the cells were seeded in 24-well tissue culture plates. The viabilities of DU145 and NE1.8 cells were determined by MTT (Sigma) assay. The viabilities of NCI-H660 cells were determined by WST-1 assay (Cayman Chemical).

### Luciferase assay

The wildtype (wt) or mutant (mut) lncRNA-p21 promoters were constructed into PGL3-basic plasmid (Promega). C4-2 and PC3 cells were plated in 24-well plates and transfected with PGL3-luc containing wildtype or mutant lncRNA-p21 promoters using Lipofectamine (Invitrogen) and pRL-TK was used as internal control. Luciferase activity was measured by Dual-Luciferase Assay reagent (Promega) based on the manufacturer’s manual.

### Chromatin immunoprecipitation (ChIP)

Briefly, protein-DNA complexes were cross-linked by 1% formaldehyde then quenched using 125 mM glycine. Cells were collected in lysis buffer and subjected to sonication. After centrifugation, the supernatant was incubated with 4 µg AR, FOXA1 or H3K4me3 antibodies, and chromatin DNA was purified and subjected to qPCR detection.

### RNA immunoprecipitation (RIP)

Briefly, C4-2 cells after different treatments were fixed by 4% paraformaldehyde and cells were lysed in ice-cold lysis buffer supplemented with RNase inhibitor. After centrifugation, 10 mg of the supernatant was cleared by protein A/G beads for 1 h and incubated with 4 µg EZH2 antibody overnight at 4 °C. The RNA was extracted using Trizol (Invitrogen) according to the manufacturer’s protocol and subjected to qRT-PCR analysis.

### Immunofluorescence staining

C4-2, C4-2 pLKO and C4-2 shlncRNA-p21 cells were seeded in the chamber slides (Thermo Fisher), and then treated with 10 µM Enz for 4 days. The cells were fixed by 4% paraformaldehyde. The primary antibodies of STAT3 and EZH2 were used at dilution of 1:200. The primary antibodies were recognized by Alexa Fluor Secondary Antibodies (Thermo Fisher, 1:1000). The images were captured by the Olympus FV1000 laser scanning confocal microscope. The Pearsons coefficient index for co-localization of STAT3 and EZH2 was analyzed by Olympus Fluoview (Olympus).

### In vivo mouse model PDX implantation and different compound treatments

The PCa-133 PDX sample was the gift from Dr. Sankar N. Maity. The PCa-133 fragments were implanted into SCID mice subcutaneously. After average tumor volumes reached 200 mm^3^, we randomly divided mice into 4 groups for i.p injections of the control DMSO, Enz (30 mg/kg) and/or EZH2 inhibitor-Dznep (1 mg/kg) every other day. Before every injection, the tumor volumes were measured. After 10 injections, the mice were sacrificed and the tumors were collected for RNA extraction, WB and IHC. All the mice were purchased from NCI. All experiments were conducted after approval from the University of Rochester Medical Center and followed the regulations of the University Committee on Animal Resources (UCAR).

### CWR22RV1 cell xenograft implantation into anterior prostate

CWR22RV1-pWPI or CWR22RV1-oelncRNA-p21 cells (1 × 10^6^) were implanted into the nude mice arterior prostate. After 5 weeks implantation, the mice were divided into 4 groups for i.p injection of DMSO and Dznep (1 mg/kg) every other day: 1. CWR22RV1-pWPI + DMSO; 2. CWR22RV1-pWPI + Dznep; 3. CWR22RV1-oelncRNA-p21 + DMSO; 4. CWR22RV1-oelncRNA-p21 + Dznep. After 10 injections, the mice were sacrificed and the tumors were collected for IHC staining. All the mice were purchased from NCI. All experiments were conducted after approval from University of Rochester Medical Center and follow the regulations of University Committee on Animal Resources.

### Immunohistochemistry staining

All of the xenografted tumors were fixed in 4% neutral buffered paraformaldehyde overnight and embedded in paraffin. The primary antibodies of SYP, ChgA, p-STAT3, p-EZH2 were used at dilution of 1:100, and the primary antibodies of ki67 and H3K27me3 were used at dilution of 1:200 for staining. The biotinylated secondary antibody (Vector) were diluted to 1:750 for recognizing primary antibodies. The signals were visualized by VECTASTAIN ABC peroxidase system and peroxidase substrate DAB kit (Vector).

### Statistics

All experiments were performed in triplicate and at least 3 times. The data values were presented as the mean ± SEM. Differences in mean values between two groups were analyzed by two-tailed Student’s *t-*test and ANOVA. *p* ≤ 0.05 was considered statistically significant.

### Reporting summary

Further information on research design is available in the Nature Research Reporting Summary linked to this article.

## Supplementary information

Supplementary Information

Description of Additional Supplementary Files

Supplementary Data 1

Supplementary Data 2

Reporting Summary

## Data Availability

All the data are available in the article and Supplementary Files, or available from the authors upon request.
