# Supplementary material for: LncRNA-p21 alters the antiandrogen enzalutamide-induced prostate cancer neuroendocrine differentiation via modulating the EZH2/STAT3 signaling
Source: Nat Commun. 2019 Jun 12;10:2571. doi: 10.1038/s41467-019-09784-9 (PMC6561926; doi:10.1038/s41467-019-09784-9)
Supplement: Supplementary file 1 — Supplementary Information [file 41467_2019_9784_MOESM1_ESM.pdf]

Supplementary Information

LncRNA-p21 alters the antiandrogen enzalutamide-induced prostate cancer neuroendocrine differentiation *via* modulating the EZH2/STAT3 signaling

Luo et al

Supplementary Figure. 1

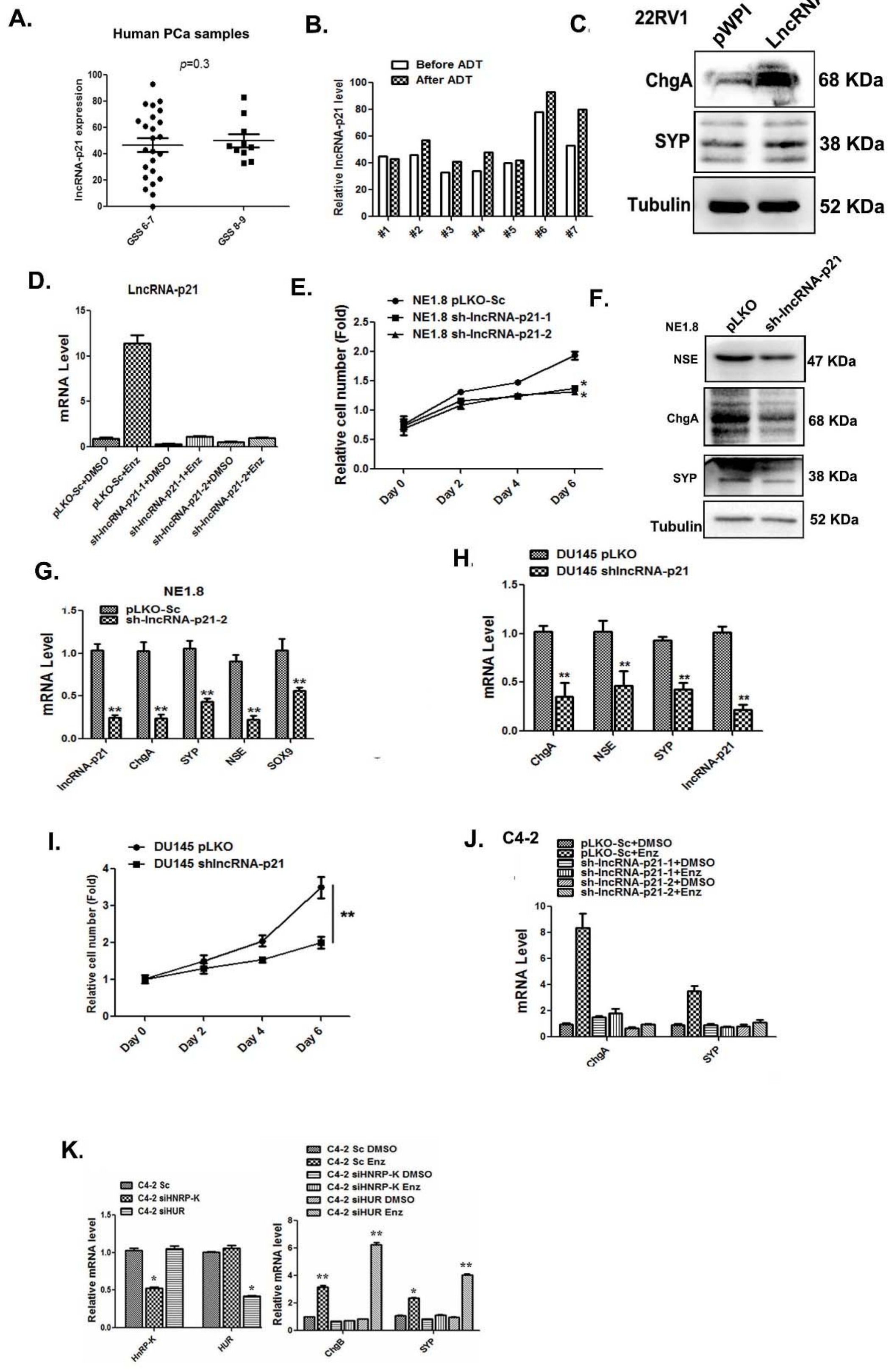

Supplementary Figure.1 LncRNA-p21 regulates NED in PCa cells

(A) The lncRNA-p21 expression in the prostate cancer tissues with Gleason score 6-7 (n=24) and Gleason score 8-9 (n=10). (B) The lncRNA-p21 expression in each patient's sample before and after ADT. (C) 22RV1 cells were infected by PWPI and PWPI-lncRNA-p21 virus. And then the NE markers expressions were analyzed by WB. (D) The lncRNA-p21 was knocked down by two shRNAs (sh-lncRNA-p21-1 and sh-lncRNA-p21-2) and the expression was detected in C4-2 cells before and after Enz treatment. (E) The lncRNA-p21 was knocked down in NE1.8 cells. The cell viability was analyzed by MTT. (F) The NE markers in NE1.8 cells after knocking down lncRNA-p21 were determined by WB. (G) The lncRNA-p21 was knocked down by sh-lncRNA-p21-2 in NE1.8 cells. And NE markers expressions were detected by qPCR. (H) The lncRNA-p21 was knocked down by sh-lncRNA-p21-2 in DU145 cells. And NE markers expressions were detected by Qpcr. (I)The lncRNA-p21 was knocked down in DU145 cells. The cell viability was analyzed by MTT. (J)The lncRNA-p21 was knocked down by two shRNAs in C4-2 cells, and then the NE markers expressions were analyzed w/o Enz treatment. (K) HNRP-K and HUR was knocked down in C4-2 cells. And the cells were treated with enzalutamide for 4 days, the NE markers expression was detected by Qpcr. For E, G, H, I and K, data are presented as mean ± SD, * $p<0.05$ ** $p<0.005$. by t-test for two groups or ANOVA for more than two groups.

Supplementary Figure. 2

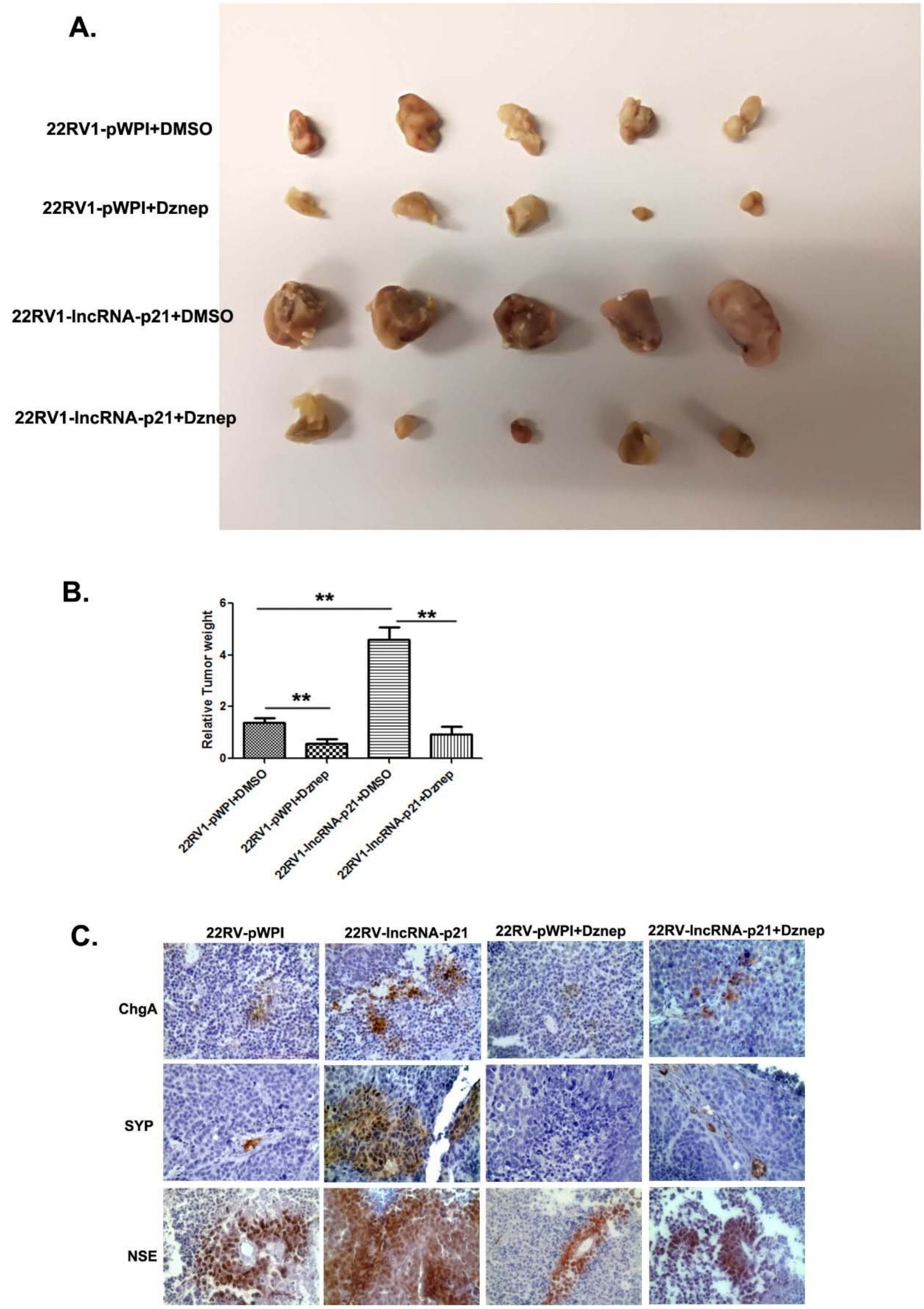

Supplementary Figure. 2 LncRNA-p21 promotes 22RV1 cell NED *in vivo*

(A) 1 million of 22RV1-pWPI and 22RV1-lncRNA-p21 cells were implanted into the anterial prostate of nude mice. After 5 weeks, the mice were randomly separated into 4 groups, each group 5 mice: 1) 22RV1-Pwpi+DMSO; 2) 22RV1-Pwpi+1ug/kg Dznep; 3) 22RV1-lncRNA-p21+DMSO; 4) 22RV1-lncRNA-p21+1ug/kg Dznep. The mice received DMSO or Dznep administration every other day. After 10 times injections, the mice were sacrificed and the tumors were collected. (B) The tumors weight of 4 groups of mice were measured. (C) The IHC staining of the NE markers expression in different groups of xenograft tumors. (Scale bar = 20μM) For B, data are presented as mean ± SD, ** p<0.005. by t-test for two groups or ANOVA for more than two groups.

Supplementary Figure. 3

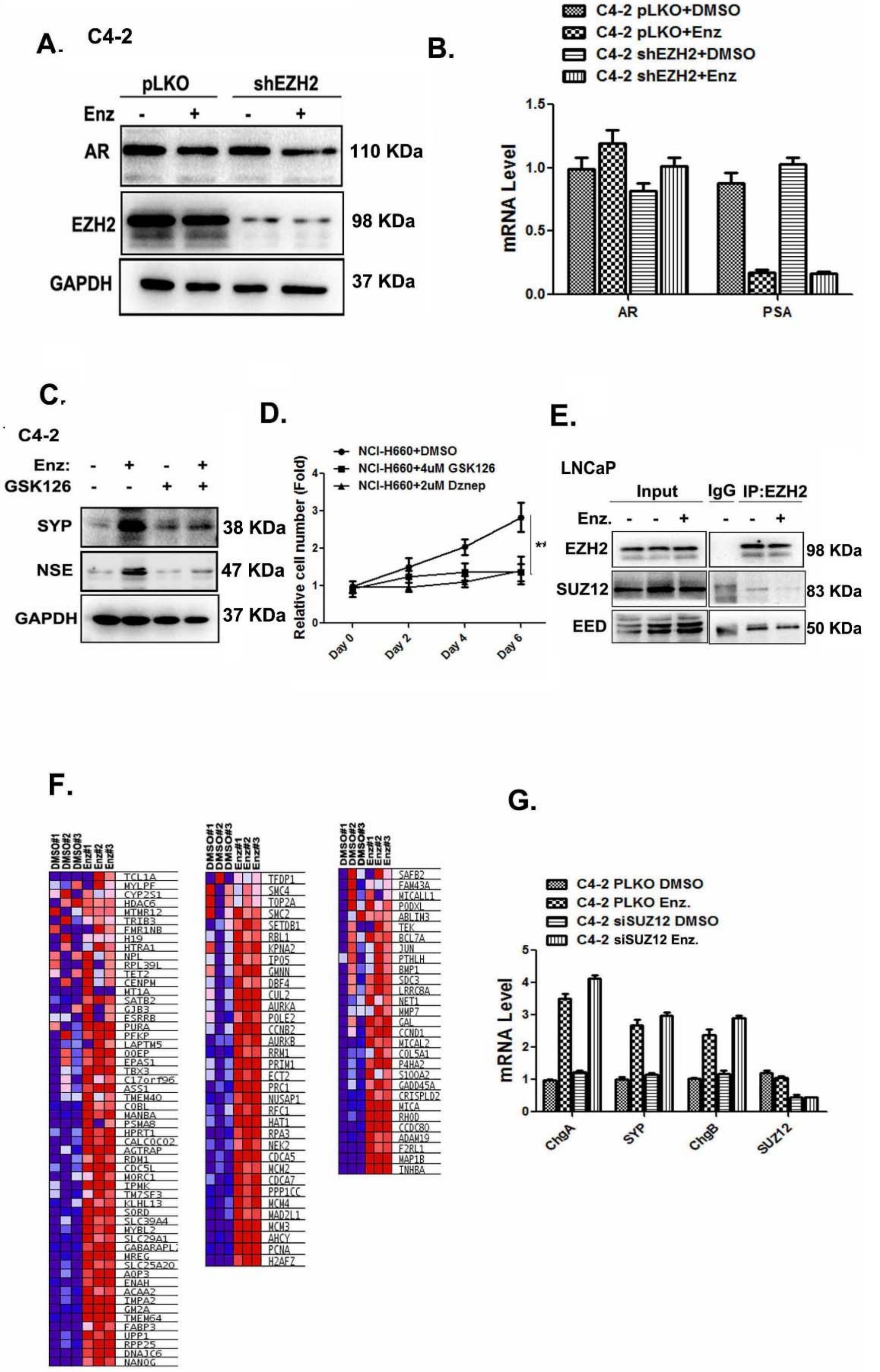

Supplementary Figure.3 Enz induces NED *via* EZH2 signal

(A) C4-2 pLKO and shEZH2 cells were treated with Enz and then the AR level was examined by WB. (B) C4-2 pLKO and shEZH2 cells were treated with Enz and the mRNA level of AR and PSA were analyzed by qPCR. (C)C4-2 cells were treated with Enz and GSK126. The NE marker expressions were detected by WB. (D) The NCI-H660 cells were treated with 4uM GSK126 or 2uM Dznep, the cell growth was analyzed by MTT. (E) LNCaP cells were treated with Enz for 4 days. The co-IP assay was performed to identified the SUZ12 and EED interaction with EZH2. (F) The heat map of the PRC2 and EZH2 target genes expression in C4-2 and Enz treated cells. (G) C4-2 PLKO and siSUZ12 cells were treated with Enz. And then the NE markers expressions were analyzed by qPCR. For D, data are presented as mean ±SD, ** $p<0.005$. by ANOVA.

Supplementary Figure. 4

A.

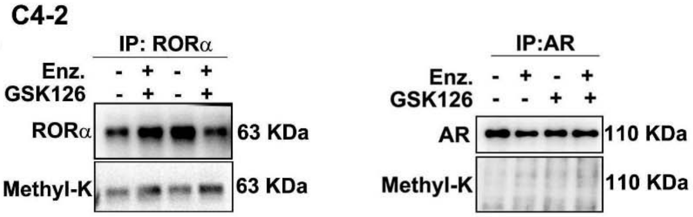

B.

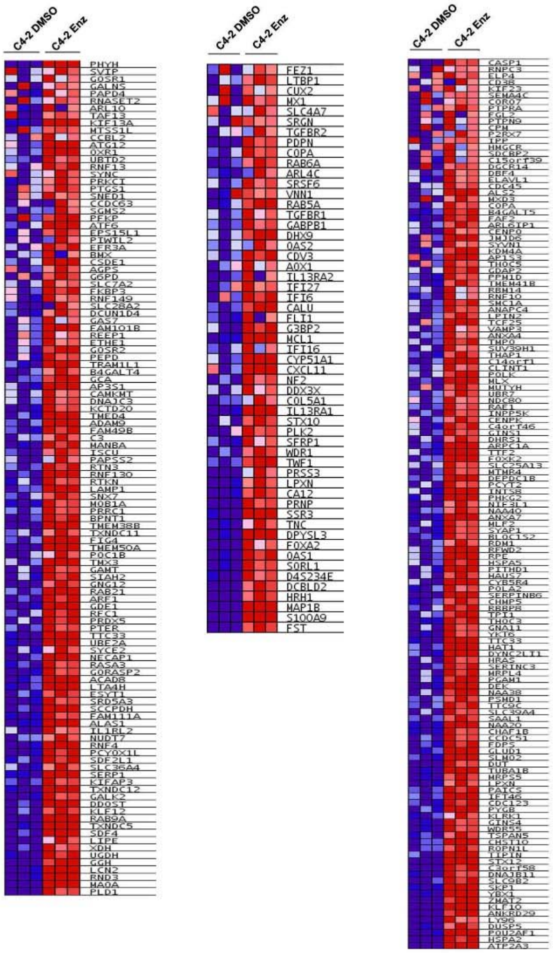

C.

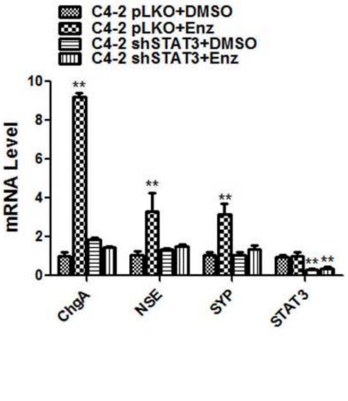

Supplementary Figure.4 Enz induces NED dependent on STAT3 signal

(A) C4-2 cells were treated with enzalutamide and GSK126 for 4 days. And then AR and RORa was pulled down and the methylation status was analyzed by WB. (B) The STAT3 target genes' expression after Enz treatment. (C) The C4-2 pLKO and shSTAT3 cells were treated w/o Enz for 6 days. The NE markers expressions were examined by qPCR. For C, data are presented as mean ± SD, ** $p<0.005$. by t-test for two groups or ANOVA for more than two groups.

Supplementary Figure. 5

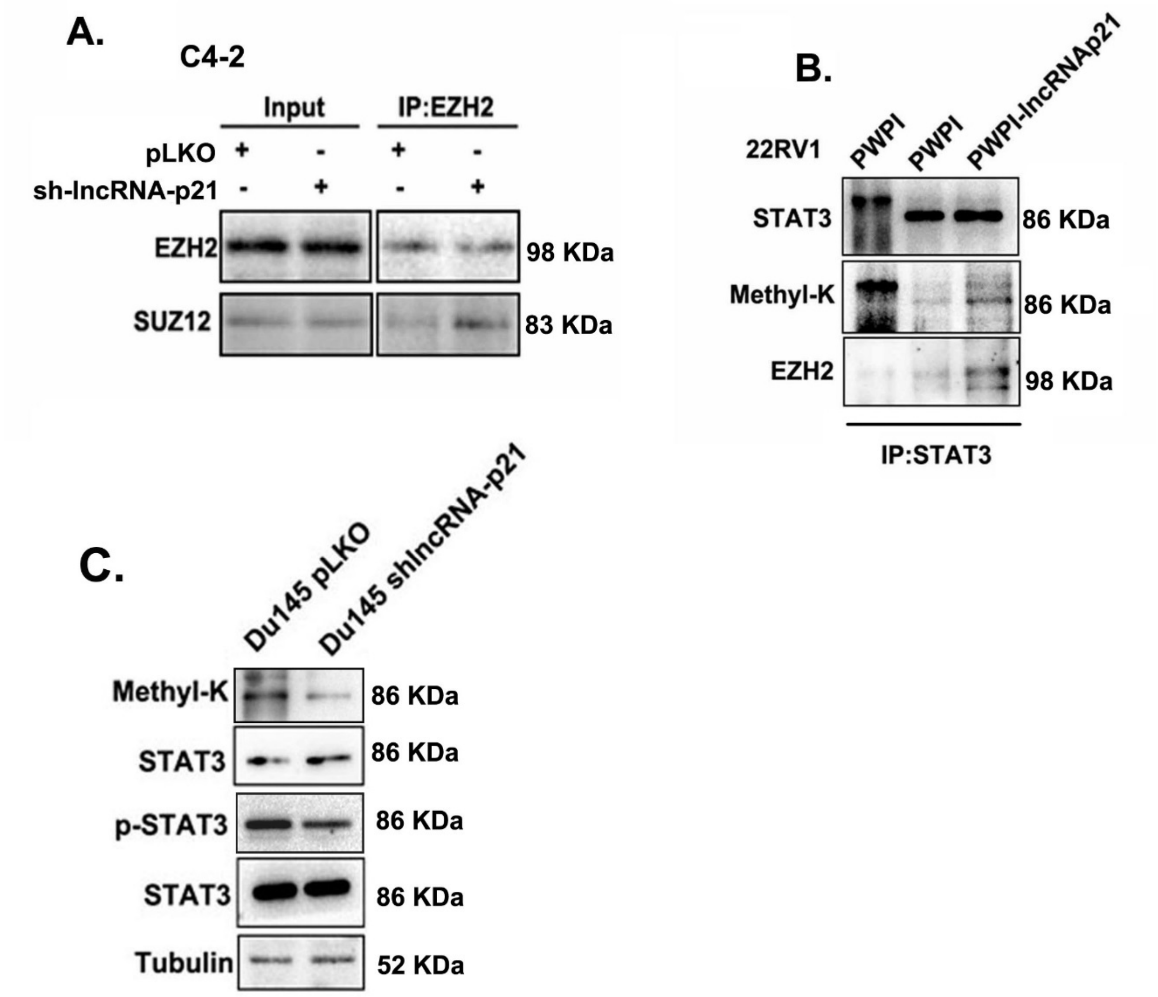

Supplementary Figure.5 LncRNA-p21 regulates EZH2/STAT3 signal

(A) The EZH2 and SUZ12 interaction was detected in C4-2 PLKO and sh-lncRNA-p21 cells. (B) LncRNA-p21 was overexpressed in 22RV1 cells, and then the STAT3 methylation and interaction between STAT3 and EZH2 was analyzed by WB (C) Du145 cells were infected by shlncRNA-p21 viruses. The STAT3 methylation and p-STAT3 levels were analyzed by WB.

Supplementary Figure. 6

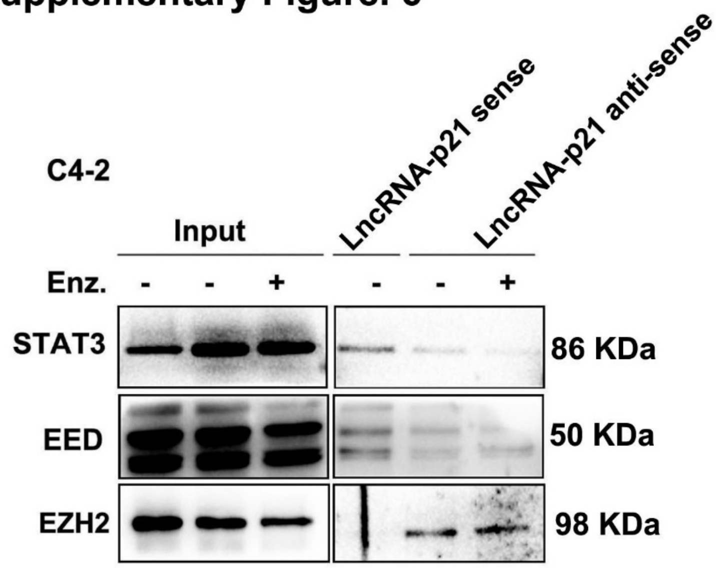

Supplementary Figure.6 LncRNA-p21 cannot interact with STAT3

C4-2 cells were treated with Enz for 4 days. And then lncRNA-p21 was pull down, the interaction between lncRNA-p21, STAT, EZH2 and EED was detected by WB.

Supplementary Figure. 7

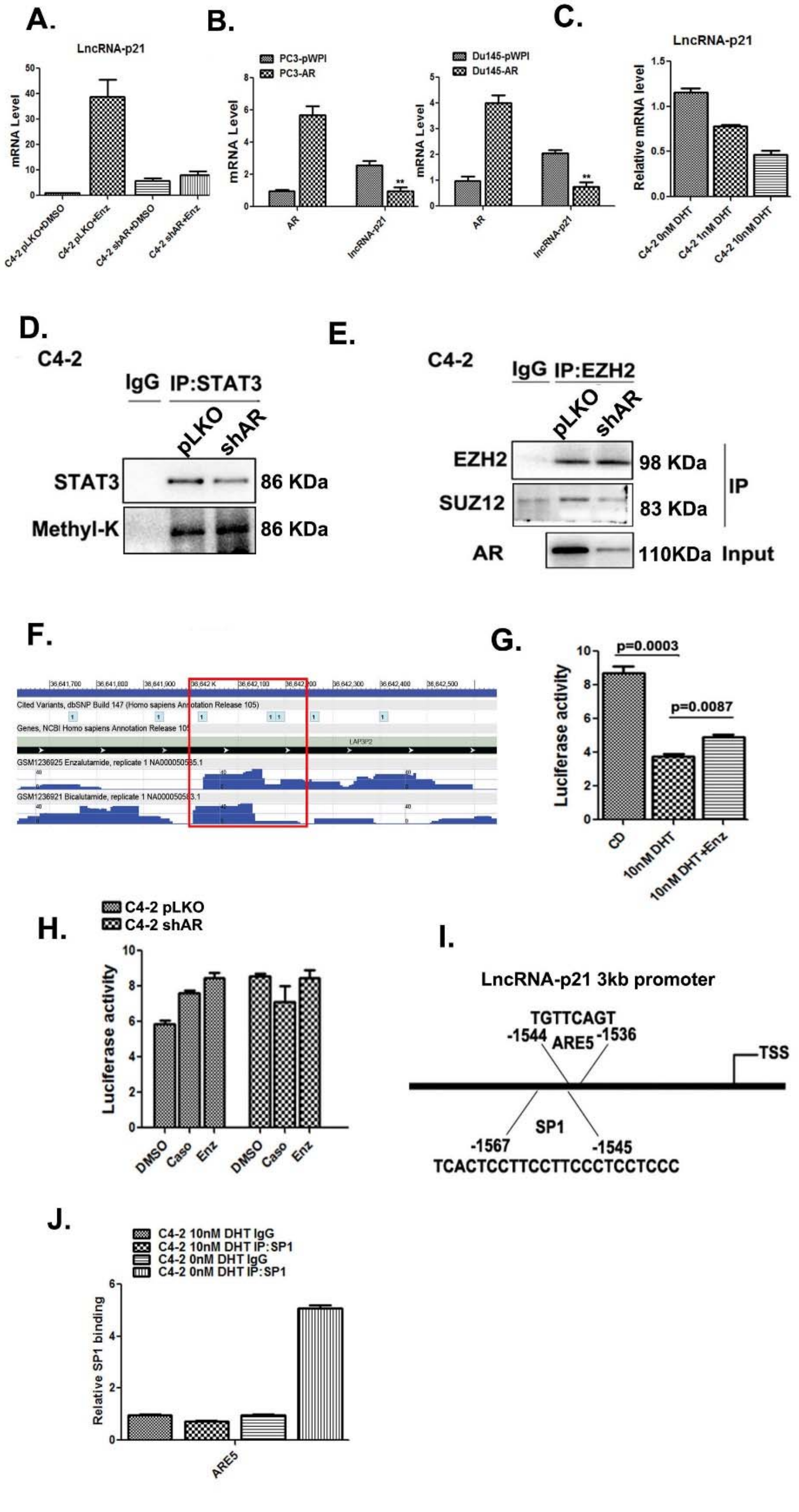

Supplementary Figure.7 AR regulates lncRNA-p21 *via* transcriptional regulation

(A) The lncRNA-p21 expression in C4-2 pLKO and C4-2 shAR cells after Enz treatments. (B) The PC3 and Du145 cells overexpressed AR, and then the lncRNA-p21 levels were analyzed by the qPCR (C) The lncRNA-p21 level decrease after DHT treatment. The C4-2 cells were cultured in CD medium 1 day, and then the cells were treated with EtOH, 1nM DHT and 10nM DHT for 2 days. The lncRNA-p21 level was detected by qPCR. (D) STAT3 methylation status was analyzed in C4-2 PLKO and siAR cells. (E) EZH2 and SUZ12 interaction was detected in C4-2 PLKO and siAR cells. (F) The ChIP-seq result of AR binding on lincRNA-p21 promoter region after Enz and Casodex treatment (GEO accession numbers: GSE43791). (G) The luciferase assay of lncRNA-p21 promoter after DHT and Enz treatment in C4-2 cells. (H) The luciferase assay of lncRNA-p21 promoter after Enz and Casodex treatment in C4-2 PLKO and siAR cells. (I) Schematic depiction of SP1REs on lncRNA-p21 promoter region. (J) ChIP assay to detect the SP1 binding on ARE5 region after DHT treatment in C4-2 cells.

Supplementary Figure. 8

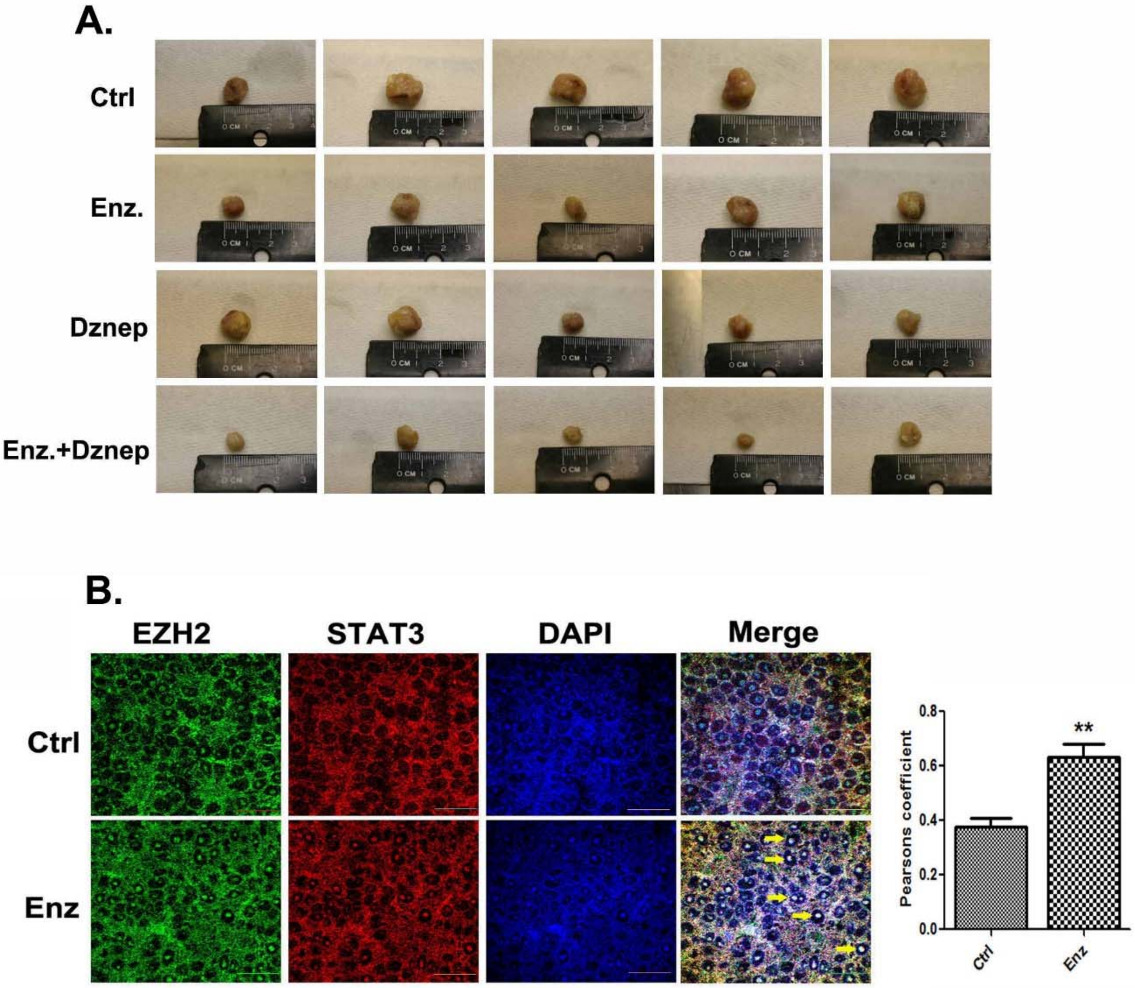

Supplementary Figure.8 PDX mouse model study

(A) The PCa-133 PDX samples were implanted into the SCID mice. After tumor sizes reached to $200mm^3$, the enzalutamide, Dznep and Vehicle were I.P injected every other day. After 10 times injection, the mice were euthanized and tumors were collected. (B) Confocal microscopic images of co-localization of EZH2 and STAT3 in the control and Enz treated PDX. The quantitation result is on right panel. The co-localization is analyzed by pearson coefficient. (** p<0.005) (Scale bar=2 um) For B, data are presented as mean ± SD, ** p<0.005. by t-test for two groups.

Supplementary Figure. 9

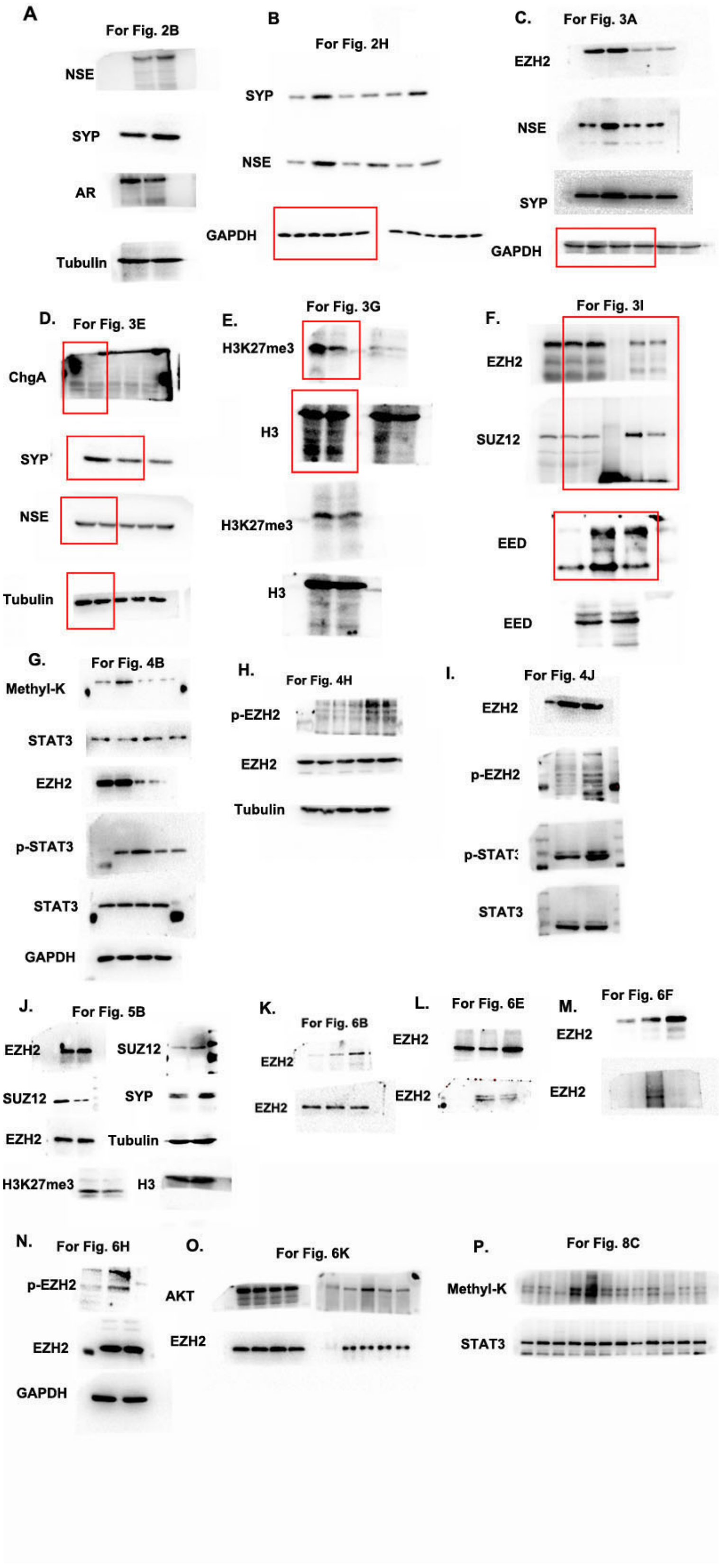

Supplementary Figure. 9 The uncropped blots for important data

(A**)** Uncropped blots for Fig. 2B. (B) Uncropped blots for Fig.2H. (C) Uncropped blots for Fig.3A. (D) Uncropped blots for Fig.3E. (E) Uncropped blots for Fig.3G. (F) Uncropped blots for Fig.3I. (G) Uncropped blots for Fig.4B. (H) Uncropped blots for Fig.4H. (I) Uncropped blots for Fig.4J. (J) Uncropped blots for Fig.5B. (K) Uncropped blots for Fig.6B. (L) Uncropped blots for Fig.6E. (M) Uncropped blots for Fig.6F. (N) Uncropped blots for Fig.6H. (O) Uncropped blots for Fig.6K. (P) Uncropped blots for Fig.8C
