# Supplementary material for: LncRNA-p21 alters the antiandrogen enzalutamide-induced prostate cancer neuroendocrine differentiation via modulating the EZH2/STAT3 signaling
Source: Nat Commun. 2019 Jun 12;10:2571. doi: 10.1038/s41467-019-09784-9 (PMC6561926; doi:10.1038/s41467-019-09784-9)
Supplement: Supplementary file 2 — Description of Additional Supplementary Files [file 41467_2019_9784_MOESM2_ESM.pdf]

Description of Additional Supplementary Files

File Name: Supplementary Data 1
Description: The expression of different lncRNAs in PCa adenocarcinoma and NEPC PDX samples.

File Name: Supplementary Data 2
Description: The list of the sequences for all oligos.
