# Supplementary material for: LncRNA-p21 alters the antiandrogen enzalutamide-induced prostate cancer neuroendocrine differentiation via modulating the EZH2/STAT3 signaling
Source: Nat Commun. 2019 Jun 12;10:2571. doi: 10.1038/s41467-019-09784-9 (PMC6561926; doi:10.1038/s41467-019-09784-9)
Supplement: Supplementary file 5 — Reporting Summary [file 41467_2019_9784_MOESM5_ESM.pdf]

nature research

Corresponding author(s): Chawnshang Chang

Last updated by author(s): 2019/03/08

Reporting Summary

Nature Research wishes to improve the reproducibility of the work that we publish. This form provides structure for consistency and transparency in reporting. For further information on Nature Research policies, see Authors & Referees and the Editorial Policy Checklist.

Statistics

For all statistical analyses, confirm that the following items are present in the figure legend, table legend, main text, or Methods section.

n/a	Confirmed	
☐	☒	The exact sample size (*n*) for each experimental group/condition, given as a discrete number and unit of measurement
☐	☒	A statement on whether measurements were taken from distinct samples or whether the same sample was measured repeatedly
☐	☒	The statistical test(s) used AND whether they are one- or two-sided<br>*Only common tests should be described solely by name; describe more complex techniques in the Methods section.*
☐	☒	A description of all covariates tested
☐	☒	A description of any assumptions or corrections, such as tests of normality and adjustment for multiple comparisons
☐	☒	A full description of the statistical parameters including central tendency (e.g. means) or other basic estimates (e.g. regression coefficient) AND variation (e.g. standard deviation) or associated estimates of uncertainty (e.g. confidence intervals)
☐	☒	For null hypothesis testing, the test statistic (e.g. *F*, *t*, *r*) with confidence intervals, effect sizes, degrees of freedom and *P* value noted<br>*Give P values as exact values whenever suitable.*
☒	☐	For Bayesian analysis, information on the choice of priors and Markov chain Monte Carlo settings
☒	☐	For hierarchical and complex designs, identification of the appropriate level for tests and full reporting of outcomes
☒	☐	Estimates of effect sizes (e.g. Cohen's *d*, Pearson's *r*), indicating how they were calculated

Our web collection on statistics for biologists contains articles on many of the points above.

Software and code

Policy information about availability of computer code

Data collection	We used the Bio-rad Image-lab 4.0.1 for collecting WB data. We used Bio-rad CFX manager 3.0 for collecting QPCR data. We used Olympus Fluoview VER1.7a for collecting confocal image data.
Data analysis	We used Olympus Fluoview VER1.7a for analyzing confocal image data. All of the statistical analysis were performed by GraphPad Prism 5.

For manuscripts utilizing custom algorithms or software that are central to the research but not yet described in published literature, software must be made available to editors/reviewers. We strongly encourage code deposition in a community repository (e.g. GitHub). See the Nature Research guidelines for submitting code & software for further information.

Data

Policy information about availability of data

All manuscripts must include a data availability statement. This statement should provide the following information, where applicable:

- Accession codes, unique identifiers, or web links for publicly available datasets
- A list of figures that have associated raw data
- A description of any restrictions on data availability

All of the data are accessible in the manuscript. The WB raw data is attached in Supplementary Figure 10.

Field-specific reporting

Please select the one below that is the best fit for your research. If you are not sure, read the appropriate sections before making your selection.

☒ Life sciences ☐ Behavioural & social sciences ☐ Ecological, evolutionary & environmental sciences

For a reference copy of the document with all sections, see nature.com/documents/nr-reporting-summary-flat.pdf

Life sciences study design

All studies must disclose on these points even when the disclosure is negative.

Sample size	The determination of the sample size is based on the previous experience. Based on the appropriate sample size, the results can reach the statistical significance.
Data exclusions	No data were excluded.
Replication	All of the experiments were repeated for at least 3 times.
Randomization	We implanted the PDX tumors or 22RV1 cells into the mice. After the tumor size reaching to the certain level, We randomly separated the 5 mice to each group for following experiments.
Blinding	The investigators were blinded to the group allocation.

Reporting for specific materials, systems and methods

We require information from authors about some types of materials, experimental systems and methods used in many studies. Here, indicate whether each material, system or method listed is relevant to your study. If you are not sure if a list item applies to your research, read the appropriate section before selecting a response.

Materials & experimental systems

n/a	Involved in the study	
☐	☒	Antibodies
☐	☒	Eukaryotic cell lines
☒	☐	Palaeontology
☐	☒	Animals and other organisms
☒	☐	Human research participants
☒	☐	Clinical data

Methods

n/a	Involved in the study	
☒	☐	ChIP-seq
☒	☐	Flow cytometry
☒	☐	MRI-based neuroimaging

Antibodies

Antibodies used	STAT3 (sc-482), AR (sc-816), GAPDH (sc-47724), tubulin (sc-23948), ChgA (sc-1488) ,and SYP (sc-17750) and ki67 (sc-23900) antibodies were from Santa Cruz Biotechnology, Inc (Santa Cruz, CA). H3K27me3 (#9733), H3K4me3 (#9751), EZH2 (#5246) and p-STAT3 (#9145) antibodies were from Cell Signaling Technology, Inc (Danvers, MA). The AKT (A01486), FOXA1 (EB05999), NSE (AP2780a), H3K27me3, H3 (620-360), Methyl-K (SPC-158F), and EED (A5371) and SUZ12 (AP20347b) antibodies were from OWL, Inc (San Diego, CA) and p-EZH2 antibody (IHC-00388) from Bethyl Laboratorys, Inc (Montgomery, TX). ROR[illegible]GTX100029[illegible] antibody were from GeneTex (Irvine, CA).
Validation	All of the antibodies are suitable for the WB, IF and IHC staining. All of the antibodies are suitable for human samples.

Eukaryotic cell lines

Policy information about cell lines

Cell line source(s)	NCI-H660 (CRL-5813), DU145 (HTB-81), PC-3 (CRL-1435), LNCaP (CRL-1740), 293T (CRL-1573) and CWR22RV1 (CRL-2505) cell lines were purchased from the American Type Culture Collection (ATCC, Manassas, VA). The C4-2 cell line was a gift from Dr. Leland W.K Chung from Cedars-Cinai, and NE1.8 cell line was a gift from Dr. Ming-Fong Lin from University of Nebraska.
Authentication	NCI-H660, DU145, PC3, LNCaP, CWR22RV1 and 293T cells were purchased from ATCC, which have been authenticated.
Mycoplasma contamination	All of the cell lines are negative for mycoplasma contamination.
Commonly misidentified lines (See ICLAC register)	None of the cells were listed in the ICLAC.

Animals and other organisms

Policy information about studies involving animals; ARRIVE guidelines recommended for reporting animal research

Laboratory animals	SCID/Ncr mouse, 6-8 weeks, male. Athymic Nude Mouse, 6-8 weeks, male.

Wild animals	The study did not involve the wild animals
Field-collected samples	The study did not involve the field-collected samples
Ethics oversight	The university of Rochester Committee on Animal Resources (UCAR) has approved the study protocol

Note that full information on the approval of the study protocol must also be provided in the manuscript.
